# VelA and LaeA are Key Regulators of *Epichloë festucae* Transcriptomic Response during Symbiosis with Perennial Ryegrass

**DOI:** 10.3390/microorganisms8010033

**Published:** 2019-12-23

**Authors:** Mostafa Rahnama, Paul Maclean, Damien J. Fleetwood, Richard D. Johnson

**Affiliations:** 1 AgResearch, Grasslands Research Centre, Palmerston North 4442, New Zealand; Paul.Maclean@agresearch.co.nz (P.M.); Damien@biotelliga.com (D.J.F.); 2 School of Biological Sciences, University of Auckland, Auckland 1010, New Zealand; 3 Biotelliga Ltd, Auckland 1052, New Zealand

**Keywords:** plant–microbe interactions, endophytes, comparative transcriptomics, velvet genes

## Abstract

VelA (or VeA) is a key global regulator in fungal secondary metabolism and development which we previously showed is required during the symbiotic interaction of *Epichloë festucae* with perennial ryegrass. In this study, comparative transcriptomic analyses of ∆*velA* mutant compared to wild-type *E. festucae*, under three different conditions (in culture, infected seedlings, and infected mature plants), were performed to investigate the impact of VelA on *E. festucae* transcriptome. These comparative transcriptomic studies showed that VelA regulates the expression of genes encoding proteins involved in membrane transport, fungal cell wall biosynthesis, host cell wall degradation, and secondary metabolism, along with a number of small secreted proteins and a large number of proteins with no predictable functions. In addition, these results were compared with previous transcriptomic experiments that studied the impact of LaeA, another key global regulator of secondary metabolism and development that we have shown is important for *E. festucae*–perennial ryegrass interaction. The results showed that although VelA and LaeA regulate a subset of *E. festucae* genes in a similar manner, they also regulated many other genes independently of each other suggesting specialised roles.

## 1. Background

*Epichloë* endophytes are symbiotic fungi that systematically colonize intercellular spaces of cool-season grasses of the subfamily Pooideae [1,2,3]. Most of these symbiotic interactions are mutually beneficial with the plant providing the endophyte with nutrients, and the endophyte protecting host plants from a range of biotic and abiotic stresses [4,5]. The bioprotective effects of *Epichloë* endophytes are mediated by in planta production of four different well-characterised classes of alkaloids: indole diterpenes, ergot alkaloids, lolines, and peramine [6].

During this mutualistic interaction, the fungus colonises most of the upper ground parts of the plant in a tightly regulated and synchronized manner [7]. Hyphae only grow in the intercellular spaces, from the meristem to the inflorescence, except for vascular bundles [2,7]. Fungal hyphae continue to grow during host cell growth, but cease growth when the host stops growing, although the hyphae remain metabolically active [8]. This results in a seldom-branched intercellular network of hyphae, parallel to the leaf axis with hyphae tightly linked with the walls of neighbouring plant cells [7]. A recent study has suggested that this hyphal network is a result of coordinated hyphal growth and death [9].

Although the molecular mechanisms that regulate the mutualistic interaction between *E. festucae* and perennial ryegrass are still largely unknown, a number of studies have shown the importance of genes required for hyphal anastomosis (soft gene [10]), fungal biology and development (*velA* gene [11] and *laeA* gene [12]), localised production of reactive oxygen species (required to maintain hyphal polarity [13,14,15,16]), and iron homeostasis (*sidN* gene [17,18]). Although it is not clear how *Epichloë* suppresses or avoids plant responses to establish and maintain a compatible interaction, recent studies have suggested that reducing the chitin-inducible host defence response by altering the fungal cell wall chitin is a possible mechanism [19,20].

Transcriptomics has also been used to investigate *Epichloë* interaction with grasses. These studies mostly focused on comparing endophyte free grasses with infected grasses [21,22,23]. In 2010, the interaction of *Epichloë festucae* with *Lolium perenne* was investigated by comparative transcriptomics of plants infected with the wild-type fungus versus plants infected with a mutant with deleted stress-activated MAP kinase gene, Δ*sakA*, by Illumina mRNA sequencing. In this study, around 11% of *E. festucae* genes were differentially expressed, with around 75% of these genes upregulated in ∆*sakA*-infected plants compared with wild-type-infected plants [24]. In 2015, the same group investigated *Epichloë festucae*/*Lolium perenne* interaction by using three fungal mutants that cause incompatible interactions in *L. perenne* [20]: ∆*proA*, which encodes a C6-Zn transcription factor that is essential for sexual fruiting body maturation in *Sordaria macrospora*; ∆*noxA*, encoding the NADPH oxidase A; and ∆*sakA*. Three comparative transcriptomic analyses of infected perennial ryegrasses with each of these mutants compared to wild-type *E. festucae*-infected plants were performed by RNA-seq. The analyses identified 182 genes that were differentially expressed in all three comparisons and these were proposed as the core fungal gene set distinguishing mutualistic from antagonistic symbiotic states [20].

The velvet gene, *veA* or *velA*, is a member of the velvet family of genes that normally includes four members, *velA*, *velB*, *velC*, and *vosA*. VelA is an important conserved fungal regulator of a variety of growth and developmental characteristics [25]. In *E. festucae*, we showed that *velA* is required for fungal biology and development and for the establishment and maintenance of the mutualistic interaction of the fungus with its host perennial ryegrass [11].

Although, based on the crystal structure and yeast one-hybrid experiments with the VosA protein, it appears that the velvet domain mediates DNA binding, the biochemical mechanism through which VelA exerts control over gene expression is not fully described [26]. Comparative transcriptomics has been used to elucidate the regulatory effects of different genes on fungal transcriptome profiles. Studies of ∆*velA* differential expression compared with wild-type fungi in different conditions have mostly used microarrays. These studies showed that VelA regulates genes involved in different fungal developmental and metabolism processes, coordinating with the functional analysis of the mutants. These processes include secondary metabolism and morphogenesis in *Penicillium chrysogenum* [27], aflatoxin biosynthetic genes in *Aspergillus flavus* [28], and fungal development and secondary metabolism in *Fusarium fujikuroi* [29]. In *Aspergillus fumigatus* and *Aspergillus nidulans*, VelA is a global regulator of secondary metabolism [30,31]. In *A. flavus*, it was shown that VelA regulates a broad range of genes, especially those involved in secondary metabolism, with 28 of 56 predicted secondary metabolite gene clusters differentially expressed [32]. In *Fusarium graminearum*, VelA is involved in regulating various cellular processes [33]. In *Botrytis cinerea*, a comparative transcriptomic analysis revealed VelA regulatory effects on fungal genes in a pathogenic interaction with its host plant *Phaseolus vulgaris*, including transporters, glycoside hydrolases, and proteases [34]. A further study of this fungus growing on solid medium showed that VelA was involved in the regulation of genes encoding secondary metabolism-related enzymes, carbohydrate-active enzymes, and proteases [35].

LaeA is a global fungal regulator and a predicted interaction partner of VelA [36] which we recently reported is required for *E. festucae* metabolism and development and for establishing and maintaining a successful symbiotic interaction with perennial ryegrass [12]. In addition, comparative transcriptomic analyses during the early stages of interaction of inoculated perennial ryegrass seedlings with the ∆*laeA* mutant and wild-type *E. festucae* suggested a regulatory role for LaeA on the expression of genes for plant cell wall degradation, fungal cell wall composition, secondary metabolism, and small secreted proteins [12,37].

Based on the knowledge of regulatory roles of VelA on different fungal transcriptome profiles and our findings of VelA importance in *E. festucae* metabolism and development and successful symbiosis [11], we hypothesised that VelA may be involved in regulating *E. festucae* transcriptome. In this study, a set of comparative transcriptomic analyses of ∆*velA* mutants versus wild-type *E. festucae* in culture, infected seedlings, and infected mature plants was performed. In addition, these results were also compared to the transcriptomic analysis of ∆*laeA* versus wild-type *E. festucae* in seedlings [12,37] and culture. We identified a specific set of VelA-regulated genes that define possible processes required for this symbiotic interaction.

## 2. Results

### 2.1. Choosing the Conditions for Transcriptomic Studies

The regulatory effects of VelA on *E. festucae* were determined using a comparative transcriptomic analysis of wild-type and ∆*velA* mutant strains grown under three different conditions; in culture, during the early stage of infection (in seedlings), and in mature infected plants (Table 1). These results were also compared to the transcriptomic analysis of ∆*laeA* versus wild-type *E. festucae* in seedlings [12,37] and culture.

In order to choose the best time for the transcriptomic study, *velA* expression was examined in wild-type *E. festucae* under the three different conditions (in culture, seedlings, and mature infected plants) employed.

As we showed previously, the highest *velA* expression in culture is under nutrient deprivation (water agar plates) and the lowest expression is in a nutrient replete medium (potato dextrose agar, PDA) [11]. Similar results were observed for *laeA* expression in different media [12]. Artificial infection of ryegrass plants with *Epichloë* was performed using fungal cultures that had been grown on PDA medium for two weeks, prior to inoculation. The same fungal cultures were used for the in-culture transcriptomic study.

**Figure 1:**
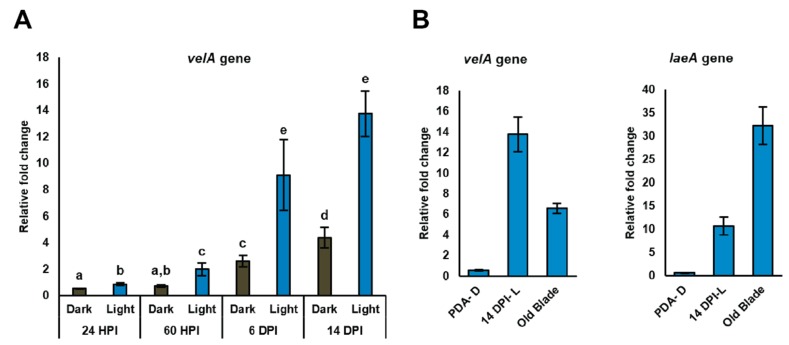
Relative expression of *velA* and *laeA* genes measured by qRT-PCR. (**A**) Relative expression of *velA* in wild-type *E. festucae* at different times after inoculating seedlings (HPI: hours post-inoculation; DPI: days post-inoculation). (**B**) Relative expression of laeA from some chosen conditions in vitro—in culture (PDA-D: potato dextrose agar under dark), seedlings (14 DPI-L: days post-inoculation under light), and mature plants (old blade). Expression values were normalised to that of gamma actin and 60S ribosomal protein L35. Primers used in the analysis are listed in Supplementary Table S1. Bars represent standard error of the mean calculated from three biological replicates. Results were analysed using the MANOVA Tukey’s test and statistically significant results are indicated with different letters.

For the seedling study, expression of the *velA* gene was examined after inoculating seedlings with wild-type *E. festucae* and growing them in both light and dark at four time points post-inoculation (24 h post inoculation (HPI), 2.5 days post inoculation (DPI), 6 DPI, and 14 DPI). The expression of *velA* increased over time and showed the highest expression at 14 DPI. At all the time points, higher expression was detected from seedlings grown under light compared to those grown under dark conditions (Figure 1A). As we showed before, the highest *laeA* expression was also at 14 DPI, although it was not a gradual increase as was the case with *velA* [12]. In addition to *velA* and *laeA* having the highest expression at 14 DPI, this time point also correlated with when ∆*velA* and ∆*laeA* mutant-inoculated seedlings began to die [11,12]. For these reasons, 14 DPI was chosen to study the requirement of *velA* and *laeA* on the expression profile of *E. festucae* during infection of perennial ryegrass.

In mature plants, as we showed before, old blades have the highest *velA* expression compared to other tissues [11] so this was also used in the current transcriptomic study.

The relative expression of *velA* and *laeA* in culture, seedlings, and mature plants was assessed using qRT-PCR. *velA* showed higher expression in seedlings compared to mature plants. This was opposite to the expression of *laeA* under the same conditions, suggesting that *velA* and *laeA* exerted different effects on the early stage of infection (Figure 1B).

### 2.2. General Description of RNA Sequencing Results

Higher numbers of reads were generated from the culture samples compared to seedlings and mature plants (Figure S1a, Table S2). Although similar numbers of reads were generated for both seedling and mature plant samples (Figure S1a), only 1.84% of total reads from mature plants mapped to the *E. festucae* genome compared to 6.28% from seedlings (Table S2), which indicated the higher fungal biomass (relative to plant tissue) in seedlings.

A multidimensional scaling (MDS) analysis of the 1000 most highly expressed genes showed that all in planta replicate samples clustered very close to each other, but were separated from the culture samples (Figure S1b).

**Figure 2:**
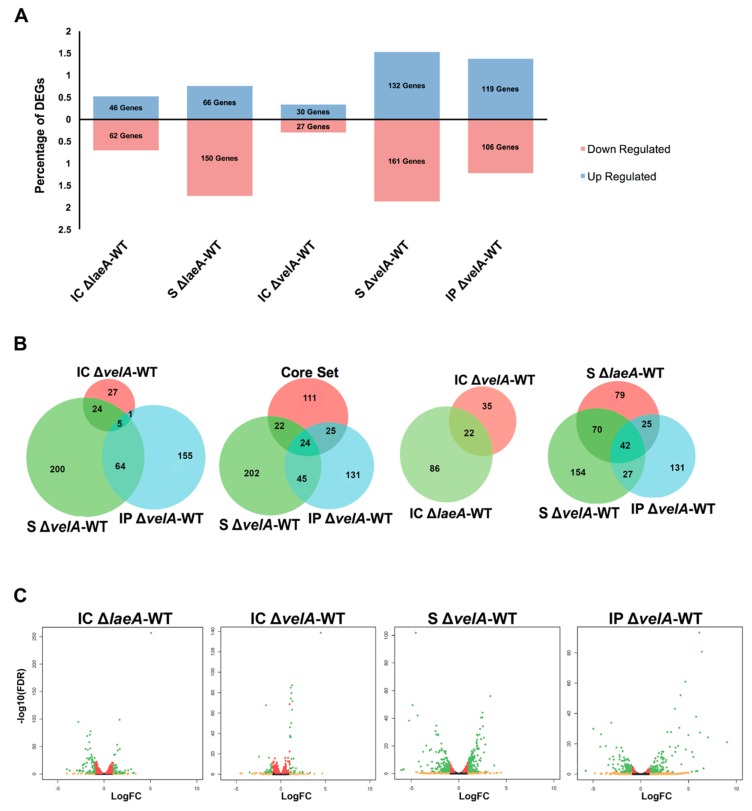
Percentage and distribution of differentially expressed genes in different comparisons and different conditions. (**A**) The bar chart shows the percentage of deferentially expressed genes (DEGs) up- or down-regulated in different comparisons. (**B**) Venn diagrams show the common DEGs between different comparisons. (**C**) Volcano plots show the distribution of log_2_ of fold changes (logFC) and–log_10_ of false discovery rate (FDR) in different comparisons. Black dots: FDR > 0.05, red dots: FDR < 0.05, orange dots: logFC > 1, green dots: FDR < 0.05 and logFC > 1.

Genes with two-fold or greater changes and an FDR (false discovery rate) equal to or less than 0.05 were counted as differentially expressed genes (DEGs) (Figure 2A). The percentages of DEGs observed in S ∆*velA*-WT were higher than that observed in other *velA*- and *laeA*-related comparisons. In S ∆*velA*-WT, 5.1 and 1.3 times more DEGs were observed compared to IC ∆*velA*-WT and IP ∆*velA*-WT, respectively, which indicated a significant impact of the growth conditions on the number of DEGs in the ∆*velA* mutant. Only 10% and 23.5% of DEGs (29 and 69 genes) in S ∆*velA*-WT were common with DEGs in IC ∆*velA*-WT and IP ∆*velA*-WT, respectively (Figure 2B).

In IC ∆*laeA*-WT, there was almost 2 times more DEGs compared to IC ∆*velA*-WT and only 15.38% (22 genes) of them were common (Figure 2A,B). DEGs in S ∆*laeA*-WT were about 26% less than S ∆*velA*-WT, but similar to IP ∆*velA*-WT (Figure 2A). More than 69% of the DEGs in S ∆*laeA*-WT were downregulated, but in both S ∆*velA*-WT and IP ∆*velA*-WT, a similar percentage of DEGs were up- and down-regulated (Figure 2A). DEGs in S ∆*laeA*-WT had 52% and 31% (112 and 67) in common with S ∆*velA*-WT and IP ∆*velA*-WT, respectively, which indicated the different regulatory effects of LaeA and VelA on the fungal transcriptome during interaction with its host (Figure 2B).

There are 182 DEGs common in the transcriptomes of three *E. festucae* mutants (∆*proA*, ∆*noxA*, and ∆*sakA*), which were proposed to form a core set of *Epichloë* genes that distinguished mutualistic from antagonistic symbiotic states [20]. Our results for S ∆*velA*-WT and IP ∆*velA*-WT showed that only 46 and 49 genes (Table S8) were in common with the proposed core set from Eaton et al. [20], respectively (Figure 2B). Thus, not all the core genes identified in the Eaton et al. [20] study are required for distinguishing mutualistic from antagonistic symbiotic states.

IP ∆*velA*-WT fold-change range (524.7 to -99.8) was 13 and 7 times greater than that of IC ∆*velA*-WT (22.3 to –11.4) and S ∆*velA*-WT (13.2 to –66.2), respectively (Figure 2C, Figure S1c). Interestingly, IC ∆*laeA*-WT (33.7 to –16.0) and S ∆*laeA*-WT (12.6 to –56.3) showed similar fold-change ranges with IC ∆*velA*-WT and S ∆*velA*-WT, respectively (Figure 2C, Figure S1c).

### 2.3. Gene Ontology (GO) Enrichment Analysis on DEGs

DEGs were classified based on their primary functions into ‘Molecular Function’ and ‘Biological Process’ GeneOntology (GO) categories (Figure S2, Table S3). It was decided that GO was not a good method to study the functions of DEGs in this study because 55%–72% of DEGs in the different comparisons were not aligned with any GO category. This had also been observed in previous studies [9,12,20], which showed that fungal genes are generally less well-characterized (many being hypothetical proteins) and are therefore not present in the GO database. Additionally, many of these genes are unique to *Epichloë*.

### 2.4. Functional Annotations of Differentially Expressed *E. Festucae* Genes

To further investigate the functions of the DEGs, BLAST analyses were performed against different databases including UniProt, Swiss-Prot, InterProScan, and KEGG and their putative functions were manually determined. In 36.8% (21), 46.1% (135), and 46.2% (104) of DEGs in IC ∆*velA*-WT, S ∆*velA*-WT, and IP ∆*velA*-WT, respectively, no significant BLAST hits (*p*-value ≤ E-20) were found. This suggested that many of the genes regulated by VelA are likely to be unique to *E. festucae*. Similar numbers of DEGs genes (35.2% (38) and 22.2% (48), respectively) were found with no significant hits in IC ∆*laeA*-WT and S ∆*laeA*-WT.

#### 2.4.1. Changes in the Expression of Genes Encoding Orthologues of Velvet Family Members

We previously reported orthologues of velvet family members in *E. festucae*: VelA (EfM3.049680), VelB (EfM3.023360), VelC (EfM3.009960), VosA or VelD (EfM3.010530), and LaeA (EfM3.069170) [11,12]. The differential expression of these genes in different comparisons showed that *laeA* expression in IC ∆*velA*-WT and S ∆*velA*-WT was upregulated. The expression of *velA* did not change significantly in ∆*laeA* mutant-related comparisons (Table S4), which suggested that whilst VelA negatively regulates the expression of the *laeA* gene, LaeA does not have any significant influence on the expression of *velA*. The expression of other members of the velvet family did not change significantly in any of the ∆*velA* or ∆*laeA* mutants (Table S4).

#### 2.4.2. DEGs in Different Functional Categories

Further analyses focussed on five areas of fungal cellular function and metabolism: fungal cell membrane transporters, fungal cell wall biosynthesis, host cell wall degradation, secondary metabolism, and small secreted proteins. These five areas included most of the DEGs that had previously been reported as important in the *E. festucae*/*L. perenne* interaction [20].

#### 2.4.3. Changes in the Expression of Genes Encoding Membrane Transporters

DEGs that encode transporters are summarised in Table 2. For the in-culture comparison, there were a small number of DEGs (4) with transporter activity, but it significantly increased (11–15) in S ∆*velA*-WT, S ∆*laeA*-WT, and IP ∆*velA*-WT. This indicated a greater regulatory effect of VelA and LaeA on the expression of *E. festucae* genes encoding membrane transporters during fungal interaction with ryegrass. Higher numbers of DEGs with transporter activity were observed in both *velA*-related comparisons (13 and 15 for S ∆*velA*-WT and IP ∆*velA*-WT) with greater fold changes (2.1–12.5) than S ∆*laeA*-WT (11 DEGs with 2–4.8 fold-change range). This suggested the stronger regulatory effects of VelA compared to LaeA on the expression of *E. festucae* membrane transporters (Table 2).

The genes with the highest fold changes (more than four times) were involved in transporting different compounds such as nitrogen, peptides, hydrogen ion, and carboxylic acid. There were two genes, EfM3.012390 and EfM3.039020, involved in nitrogen transport. EfM3.012390, which was only differentially expressed in IP ∆*velA*-WT (4.9-fold upregulated), is a homologue of a nitrate transporter, CrnA, from *Emericella nidulans* and has been shown to be involved in nitrogen metabolite repression [38]. EfM3.039020, which was upregulated in both S ∆*velA*-WT and IP ∆*velA*-WT, is a homologue of an ammonium transporter, Amt1, from *Schizosaccharomyces pombe* [39]. EfM3.027570 was upregulated in both S ∆*velA*-WT and IP ∆*velA*-WT and is a homologue of a peptide transporter, PTR2, from *Stagonospora nodorum* [40].

These results suggested that ∆*velA* and ∆*laeA* mutants may be nutrient-starved during interaction with seedlings or mature plants.

#### 2.4.4. Changes in the Expression of Genes Encoding Enzymes with Host Cell Wall-Degrading Activity

Fundamentally, plant cell walls are made of embedded cellulose microfibrils in a matrix of pectin, hemicellulose, and cell wall-associated proteins [41]. To look specifically at the enzymes with plant cell wall-degrading characteristics, the Fungal PCWDE (plant cell wall-degrading enzymes) Database [42], was utilised. In this database, there are 22 known fungal gene families which degrade the plant cell wall [42]. A search against this database showed that 25 genes in 13 PCWDE families are present in *E. festucae* (Table S5).

In addition, the larger carbohydrate-active enzyme database (CAZyme) [43], which includes the families of enzymes that assemble, modify, or breakdown oligo- and poly-saccharides, was checked [43]. The presence of 310 genes in *E. festucae* that are homologues with CAZyme families [20] were analysed for DEGs across the different comparisons (Figure S3). The number of DEGs with homologues to CAZyme families was almost ten times and two times increased in S ∆*velA*-WT compared to IP ∆*velA*-WT and IC ∆*velA*-WT, respectively, and two times increased in S ∆*laeA*-WT compared to IC ∆*laeA*-WT (Figure S3). These results indicated the importance of VelA and LaeA on the expression of CAZyme genes during *E. festucae* interaction with its host plant.

All DEGs with plant cell wall-degrading activity are summarised in Table 3. No DEGs belonging to cellulases, xylanases, cutinases, or pectinases were detected for the in-culture comparisons. No DEGs with cellulose degradation activity were detected for S ∆*velA*-WT and IP ∆*velA*-WT, but two DEGs with cellulose activity were detected in S ∆*laeA*-WT.

Three DEGs, EfM3.040190, EfM3.005420, and EfM3.037040, with hemicellulose activity were detected (Table 3). Two of them, EfM3.037040 and EfM3.040190, were upregulated in all S ∆*laeA*-WT, S ∆*velA*-WT, and IP ∆*velA*-WT comparisons, with the IP ∆*velA*-WT comparison showing the highest fold change of all DEGs with plant cell wall degradation activity (Table 3). EfM3.037040 is a homologue of xylanase C (XynC) from *Cellvibrio japonicus* which hydrolyses hemicellulose. This gene was 4.3-, 4.3-, and 69.6-fold upregulated in ∆*laeA*-WT, S ∆*velA*-WT, and IP ∆*velA*-WT, respectively. EfM3.040190 is a homologue of endo-1,4-beta-xylanase 2 (*xyl2*) from *Claviceps purpurea* [44] and was 3.3-, 3.9-, and 83.5-fold upregulated in S ∆*laeA*-WT, S ∆*velA*-WT, and IP ∆*velA*-WT, respectively. In addition to these two genes, another DEG was observed with hemicellulose activity, EfM3.005420, that is a homologue of exo-1,4-beta-xylosidase (*bxlB*) from *Aspergillus flavus* and which was 2.3-fold upregulated in S ∆*velA*-WT.

One DEG, EfM3.008730, was upregulated by 2-fold in S ∆*velA*-WT and encodes a pectinase with homology to pectin methyl esterase (*pme1*) in *Aspergillus aculeatus*. It has been shown that Pme1 in *A. aculeatus* degrades the host plants’ cell wall [45].

A downregulated DEG, EfM3.005300, identified from both seedlings and in planta comparison, is a homologue of the cuticle-degrading protease from the insect pathogen *Metarhizium anisopliae* [46].

Based on these data, it appears that VelA regulates *E. festucae* genes that encode proteins with a range of plant cell wall-degrading functions during the fungal interaction with its host. Of the surviving plants infected with the ∆*velA* mutant, there was a significantly greater hemicellulose degradation activity which may contribute to the observed phenotypes of mutant-infected plants [11].

#### 2.4.5. Changes in the Expression of Genes Encoding Proteins Involved in Fungal Cell Wall Composition

Chitin, glycoproteins, and glucan are the main components of the fungal cell wall and these can function as elicitors of plant defence responses [47,48]. Fungi use a range of enzymes to break down, synthesise, or remodel their cell wall [49]. All detected DEGs that encode proteins related to the fungal cell wall are summarised in Table 4.

Enzymes for the synthesis and breakdown of chitin are chitin synthases and chitinases, respectively [49]. EfM3.000810 is a homologue of a chitinase (BDCG_06828) from *Blastomyces dermatitidis* and was 3-, 3.3-, and 3.1-fold downregulated in S ∆*laeA*-WT, S ∆*velA*-WT, and IP ∆*velA*-WT, respectively. EfM3.024310 is a homologue of endochitinase B (*chiB1*) from *Neosartorya fumigata* [50] and was 2.1-fold upregulated in IP ∆*velA*-WT. EfM3.049120 is a homologue of chitin synthase 1 (*chs-1*) from *Neurospora crassa* and was 2-fold upregulated in IC ∆*velA*-WT. This gene has been shown to play a major role in cell wall biogenesis in this fungus [51].

No DEGs that encode β-1,3-glucan synthase enzymes, responsible for synthesis of fungal cell wall glucan, were found in *E. festucae* but there were two genes, EfM3.056450 and EfM3.056810, that are possibly engaged in breaking down glucan. EfM3.056450 is a homologue of EPD1 from the dimorphic yeast *Candida maltosa*. In this fungus, it was shown that EPD1 is necessary for fungal transition to the pseudohyphal growth form. Deleting this gene leads to a reduction of both alkali-soluble and alkali-insoluble β-glucan levels [52]. EfM3.056450 also has a glucanosyl transferase domain that in some cases has been shown to remodel chains of β-1,3-glucan in the fungal cell wall [53]. This gene was upregulated by 2.1-fold in IP ∆*velA*-WT. EfM3.056810 is a homologue of glucan endo-1,3-beta-glucosidase from *Cellulosimicrobium cellulans* and was 2.1-fold downregulated in IP ∆*velA*-WT. This enzyme was shown to be involved in breaking down fungal cell walls by hydrolysis of cell wall β-1,3-glucosidic linkages [54].

Glycoproteins are another constituent of the fungal cell wall that are made of modified proteins by *N*- and *O*-linked carbohydrates that in many examples are glycosyl phosphatidyl inositol (GPI) anchors [49]. There were three genes, EfM3.078790, EfM3.054000, and EfM3.034340, with possible activity towards glycoproteins (Table 4). EfM3.078790 is a homologue of a cell wall glycoprotein (*sed1*) from *Saccharomyces cerevisiae* that was differentially expressed in seedling comparisons (2.5-fold downregulated in S ∆*velA*-WT). In *S. cerevisiae*, Sed1 is the most abundant cell wall-associated protein in the stationary phase and is necessary for fungal resistance to lytic enzymes [55]. EfM3.054000 is a homologue of a putative cell wall glycoprotein (CPUR_07530) from *Claviceps purpurea* that was 2.3-fold upregulated in S ∆*velA*-WT. EfM3.034340 is a homologue of the collagen α-2 (IV) chain in the nematode *Ascaris suum* and was 3.4- and 3.7-fold downregulated in S ∆*laeA*-WT and IP ∆*velA*-WT. Although collagen is best understood in animals, it is also detected in fungal fimbriae, long (1–20 µm) and narrow (7 nm) flexuous appendages on the fungal surface which affect cellular functions, such as mating and pathogenesis [56]. This gene is also a homologue of a putative cell wall protein (XP_001270917) in *Aspergillus clavatus*.

One of the fungal phenotypes observed in mature plants infected with the mutant ∆*velA* was the formation of intrahyphal hyphae [11]. It has been shown that triggering the activation of woronin bodies, important organelles that block the septal pore in response to wounding, increases the formation of intrahyphal hyphae [57]. EfM3.049350 is a homologue of hex protein, a major woronin body protein in *Neurospora crassa*, which was upregulated by 2.9 times in IP ∆v*elA*-WT.

These results demonstrated that VelA is a regulator of *E. festucae* cell wall composition during the fungal interaction with ryegrass.

#### 2.4.6. VelA is Required for Secondary Metabolite Gene Expression and Production

In order to investigate the regulatory effects of VelA on secondary metabolism in *E. festucae*, the differential expression of genes involved in known alkaloid gene clusters (ergot alkaloids, indole diterpenes, and peramine) was examined across all comparisons. In the in-culture comparison, the *lpsB* gene from the ergovaline pathway was upregulated (more than 9-fold) in both ∆*velA* and ∆*laeA* mutants. Another gene from this pathway, *easA*, was also upregulated (2-fold) in the ∆*laeA* mutant (Table S6).

**Figure 3:**
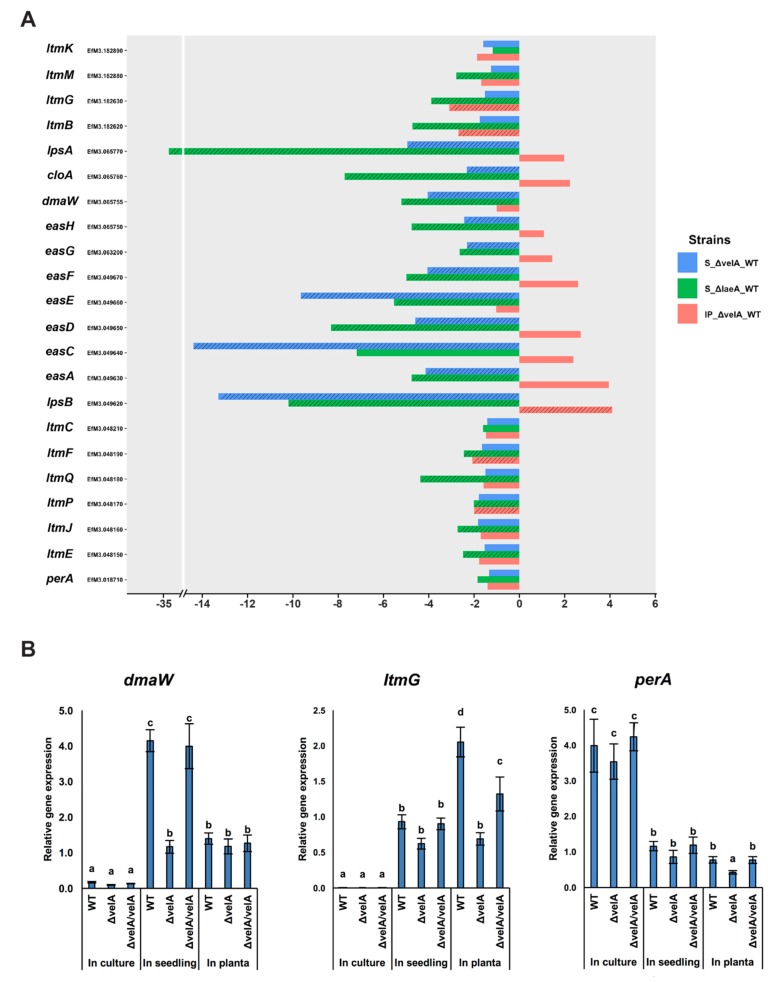
Expression changes of *E. festucae* alkaloid biosynthesis genes in different conditions. (**A**) Expression changes of *E. festucae* alkaloid biosynthesis genes in different comparisons including S ∆*laeA*-WT, S ∆*velA*-WT, and IP ∆*velA*-WT. Dashed bars: fold changes that are statistically significant (FDR ≤ 0.05), non-dashed bars: fold changes that are statistically non-significant (FDR ≥ 0.05). (**B**) Relative expression levels of three representative ergovaline, lolitrem B, and peramine biosynthetic genes in wild-type, Δ*velA*, and *ΔvelA/velA* in three different conditions. Results were analysed using the MANOVA Tukey’s test and independent sample T-test and statistically significant results are indicated with the same letter are not significantly different.

Of the 11 genes involved in ergovaline production (*EAS* cluster genes) [58], only one gene, *lpsB*, was upregulated in IP ∆*velA*-WT, whereas all genes were downregulated in S ∆*velA*-WT, which was similar to S ∆*laeA*-WT (Table S6, Figure 3A).

Of the ten known genes for indole diterpene biosynthesis, four genes were significantly downregulated in IP ∆*velA*-WT. In S ∆*velA*-WT, none of the genes were differentially expressed, which was in stark contrast to S ∆*laeA*-WT in which eight genes were downregulated. This indicated very different regulatory roles of *velA* and *laeA* genes on fungal secondary metabolite regulation in *E. festucae* (Table S6, Figure 3A).

The expression of *perA*, the sole gene required for peramine biosynthesis, was not differentially expressed in any comparison (Table S6, Figure 3A).

The transcriptomic results were validated by qRT-PCR of *dmaW*, *ltmG*, and *perA*, the first gene from the ergovaline, lolitrem B, and peramine pathways, respectively, in all three different conditions using wild-type, mutant, and complemented strains (Figure 3B). qRT-PCR results validated the results of transcriptomic analyses (Figure 3A).

**Figure 4:**
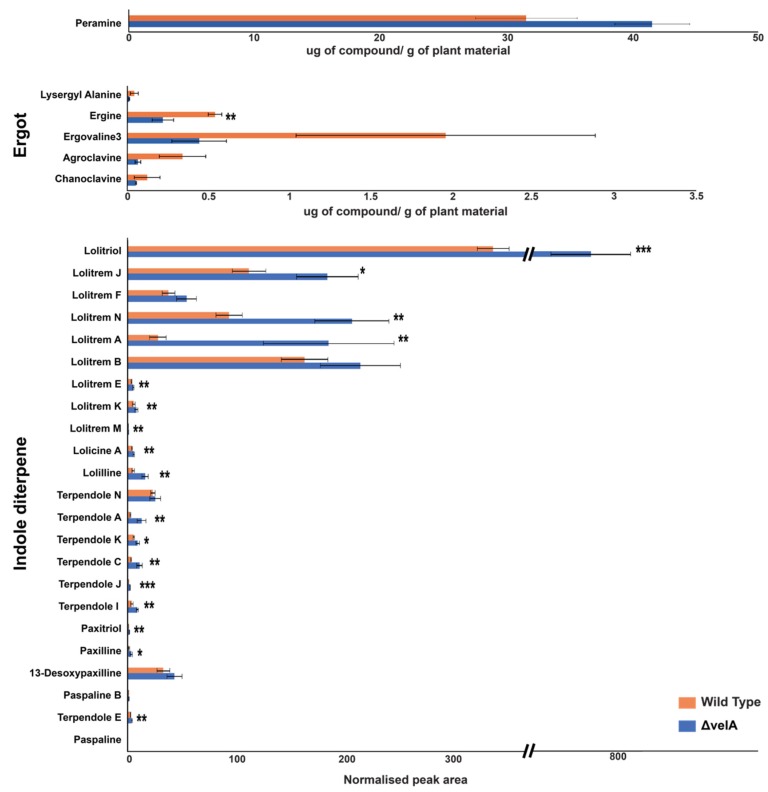
VelA regulates alkaloid production of *E. festucae* in ryegrasses plants. Alkaloids’ concentration in three-month-old ryegrasses infected with wild-type and ∆*velA* mutant *E. festucae*. Results were analysed using the independent sample T-test and statistically significant results are indicated (*** *p* < 0.001, ** 0.05 > *p* > 0.001, * 0.1 > *p* > 0.05).

To correlate alkaloid pathway gene expression results, different alkaloids concentrations were also measured in infected three-month-old ryegrass plants infected with wild-type and ∆*velA* mutant *E. festucae* (Figure 4). Of the seven ergot alkaloid compounds that were measured, two of them, lysergic acid and elymoclavine, were not detected. For the remaining five compounds, the mean concentrations in the wild-type-infected plants were higher than ∆*velA* mutant-infected plants, but only ergine was statistically significant (Figure 4). Of the 23 indole diterpene alkaloid compounds that were measured, the mean concentrations of the ∆*velA* mutant-infected plants were higher with 17 of them being statistically significant (Figure 4). For peramine, the mean concentration in ∆*velA* mutant-infected plants was also higher, but it was not statistically significant (Figure 4).

We previously reported alkaloid production in three-month-old ryegrass plants infected with wild-type and ∆*laeA* mutant *E. festucae* [12]. The differences of mean concentration of different alkaloids produced in the wild-type-infected plants and ∆*velA* mutant-infected plants were compared to the mean concentration differences of wild-type infected plants and ∆*laeA* mutant-infected plants (Figure S4). Results showed lower concentrations of most indole diterpene alkaloids in the ∆*laeA* mutant-infected plants, which was opposite to that observed for ∆*velA* mutant-infected plants (Figure S4).

**Figure 5:**
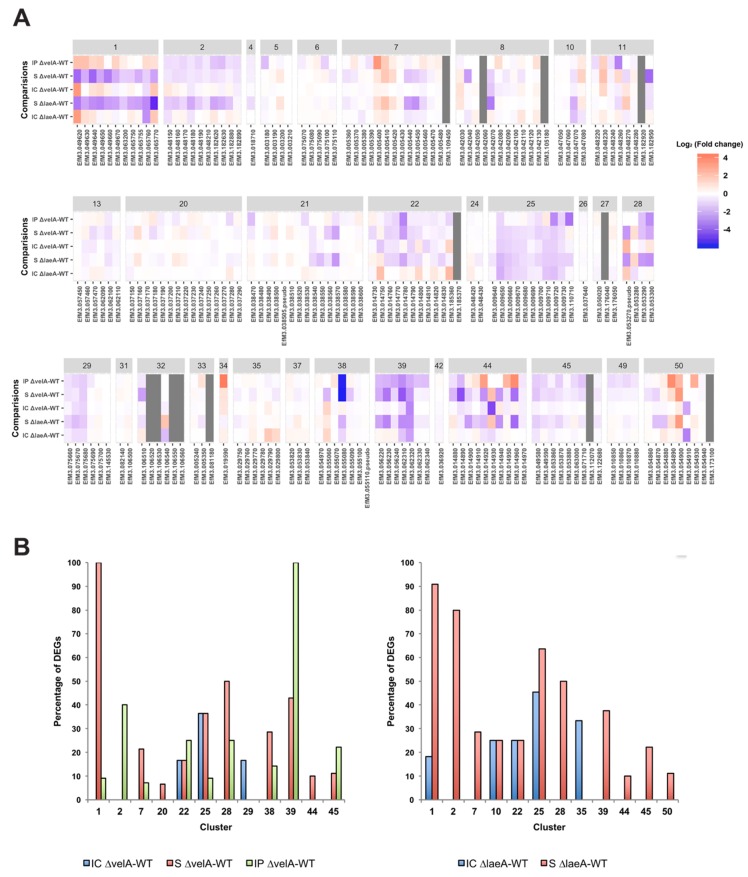
Transcriptomic profile of all secondary metabolism clusters in *Epichloë festucae*. (**A**) Heatmap shows fold changes of all genes in each secondary metabolism cluster. Grey colour cells show that in that condition none of the mutant or wild-type are expressed. (**B**) Percentage of DEGs in the clusters with at least one DEG in *velA*-related comparisons (left panel) and *laeA*-related comparisons (right panel); modified from Rahnama et al. [12] with permission.

In addition to these 3 alkaloid clusters, 29 other clusters with 191 genes, which putatively encode secondary metabolites in *E. festucae* Fl1 [6], were also examined (Table S6). Heat maps generated for all 32 clusters in *E. festucae* showed that most of them were downregulated in ∆*velA* mutant-infected plants (Figure 5A), indicating that VelA positively regulates secondary metabolite gene expression in *E. festucae*. Of the 32 clusters, 12 contained at least one gene differentially expressed in one of the ∆*velA*-related comparisons; 9 clusters in IP ∆*velA*-WT, 10 clusters in S ∆*velA*-WT, and 3 clusters in IC ∆*velA*-WT had DEGs (Figure 5B, left panel). In the ∆*laeA*-related comparisons, also 12 clusters contained at least one gene differentially expressed in one of the comparisons, 11 clusters in S ∆*laeA*-WT and 5 clusters in IC ∆*laeA*-WT (Figure 5B, right panel), but these clusters were not all identical to the ones in the ∆*velA*-related comparisons. Clusters 20, 29, and 38 were unique to ∆*velA*-related comparisons and clusters 10, 35, and 50 were unique to ∆*laeA*-related comparisons (Figure 5B).

Our results showed that VelA, similar to LaeA, is a key regulator for secondary metabolite gene expression and production in *E. festucae* and that VelA and LaeA exert different regulatory effects.

#### 2.4.7. Changes in the Expression of Genes Encoding Putative Small Secreted Proteins (SSPs)

**Figure 6:**
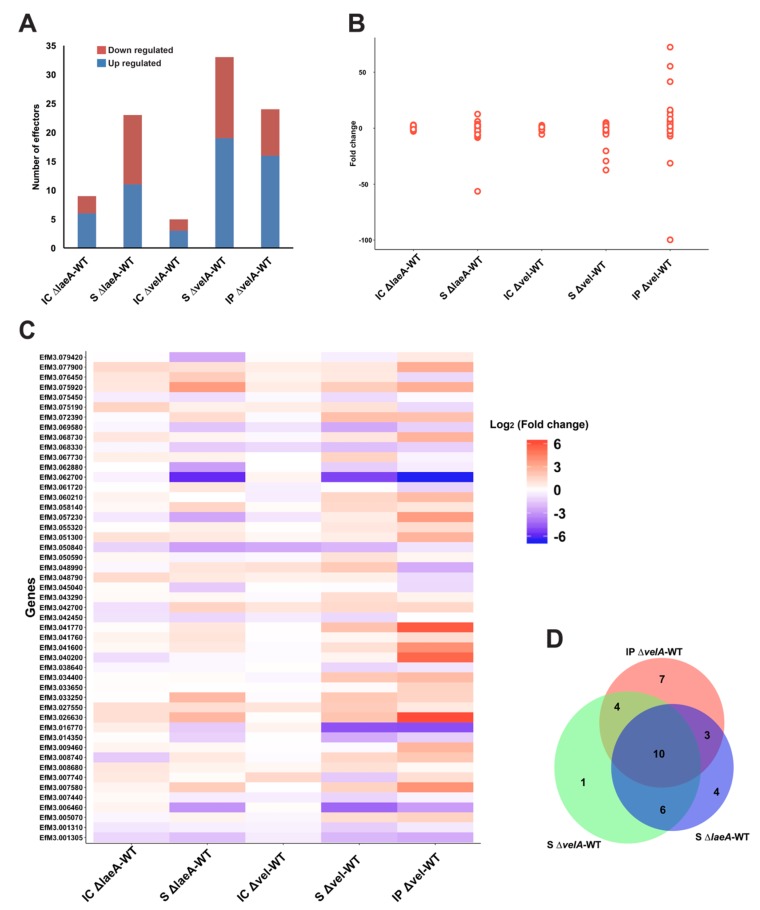
DEGs with putative small secreted proteins (SSPs) function. (**A**) Number of DEGs, down- or up-regulated, in each comparison with putative SSP functions. (**B**) Distribution of SSPs’ fold change in different conditions, each point representing one SSP. (**C**) Heat map showing the different ranges of regulatory effects of VelA and LaeA on the expression of putative SSPs in different conditions. (**D**) Venn diagram shows the common SSPs between different comparisons which are significantly differentially expressed.

Microbes during infection of host plants produce effector proteins that are of key interest during plant–microbe interactions. These secreted proteins suppress or interfere with host immune responses or alter the host cell physiology via a range of different molecular mechanisms, generating a favourable environment for infection and growth [59,60]. Recently, 141 genes in *E. festucae* were reported as putative effectors [61]. Of these, 49 were differentially expressed in at least one of the comparisons described here (Figure 6, Table S7). Although S ∆*vel*A-WT had the highest number of SSPs (Figure 6A), IP ∆*vel*A-WT had a much broader range of fold changes (from 72.4 to –99.8) (Figure 6B). *velA* and *laeA* showed different regulatory effects on SSPs across the different conditions (Figure 6C) with some being unique to particular conditions (Figure 6D).

Most of the predicted gene products have no homologues with annotated molecular function, except four genes EfM3.001305, EfM3.001310, EfM3.055320, and EfM3.079420 (Table S7). EfM3.001305 and EfM3.001310 are homologues with a killer toxin gene, *kp4*, from *Metarhizium spp* that is a lethal protein via inhibition of calcium channels [62]. EfM3.055320 was only expressed in in-planta comparisons and is a homologue of Cu/Zn superoxide dismutase from *Claviceps purpurea* [6], which is involved in reducing superoxide radicals generated by host plant defence mechanisms. EfM3.079420 is a homologue of a fungal hydrophobin domain-containing protein in *Pochonia chlamydosporia* [63].

Three putative effectors, which are some of the core symbiosis genes [20], showed the highest differential gene expression and were downregulated. EfM3.016770, EfM3.062700, and EfM3.014350 called *sspM*, *sspN*, and *sspO*, respectively, were functionally studied in Hassing et al. [61], but no clear function was identified presumably due to the redundancy functions of the effectors. These three effector candidates were also downregulated in all S ∆*velA*-WT, IP ∆*velA*-WT, and S ∆*laeA*-WT comparisons (Table S7).

Although only 27% (49 genes) of the Eaton et al. [20] symbiosis core genes were common with DEGs in the mature infected plants with ∆*velA*, 16% (8 genes) of these were SSPs, which make up 57% (8/14) of the total SSP genes in the Eaton et al. [20] core set.

## 3. Discussion

The *velA* gene is a well-known regulatory gene required for different fungal cellular and developmental functions, including secondary metabolism, pathogenicity, sexual and asexual development, and fungal morphology and growth [25,64]. In addition, we recently showed that it is required for the symbiotic interaction of *E. festucae* with perennial ryegrass [11]. In this study, by using comparative transcriptomics, VelA regulatory effects on the transcriptome profile of *E. festucae* growing in different conditions (in culture, inoculated seedlings, and mature infected plants) were tested and the results were compared with similar experiments, previously reported, on the other fungal global regulator LaeA [12,37].

The number of DEGs in S ∆*vel*A-WT and IP ∆*velA*-WT was 5.1 and 3.9 times higher than IC ∆*velA*-WT, respectively. This suggested that VelA has a stronger regulatory effect when growing in seedlings and mature plants under nutrient-limited conditions, compared to PDA culture, a nutrient-rich condition. This also correlated with our previous report that showed much higher expression of *velA* in nutrient-limited medium (water agar) compared to nutrient-rich medium (PDA) [11], which is similar to LaeA [12].

The small proportion of DEGs common between IC ∆*velA*-WT, S ∆*velA*-WT, and IP ∆*velA*-WT suggested condition-dependent regulatory roles of VelA on *E. festucae* gene expression, similar to LaeA. It is possible that VelA forms different protein complexes in different conditions as was shown in *A. nidulans* [25]. Other possibilities are different post-translational modifications or localisation under different conditions.

Compared to the core symbiotic fungal gene set identified by Eaton et al. [20], only a small proportion of DEGs from both IP ∆*velA*-WT and S ∆*velA*-WT were common. This may partly be a result of different tissue samples used for the transcriptomic analyses between the two studies. Based on the difference in hyphal in planta characteristics between mutant *velA* and *proA*, *noxA*, and *sakA* mutants, it is most likely that VelA, like LaeA, regulates separate mechanisms important in mutualistic interactions than ProA, NoxA, and SakA. One of the distinct differences between the in planta fungal growth of *velA* and *laeA* mutants, compared to *proA*, *noxA*, and *sakA* mutants was chitin distribution. In *velA* and *laeA* mutants, chitin distribution was very similar to the wild-type interaction [11,12], whereas for *proA*, *noxA*, and *sakA* mutants, chitin distribution was increased [20].

Comparative transcriptomic studies of *∆velA* mutants in different fungi showed conserved regulatory roles of VelA on secondary metabolism, CAZyme biosynthesis, morphogenesis, development, and cellular metabolism [27,28,29,30,31,32,33,35], that are quite similar with some reported roles of LaeA [35,65,66,67]. In the fungal transcriptomic analysis of S ∆*velA*-WT, IP ∆*velA*-WT, S ∆*laeA*-WT, upregulation was observed in the expression of genes that encode putative nutrient transporters, host cell wall-degrading enzymes, and small secreted proteins. In these three functional categories, DEGs in IP ∆*velA*-WT showed higher differential expression with more genes involved, compared with seedling comparisons, although total DEGs in this comparison was not higher than others. This suggested that VelA has stronger regulatory influence on fungal gene expression for these three functional categories during interaction with mature plants compared to the early stages of infection.

Fungal transporters of nitrate, ammonium, peptides, and carboxylic acid were upregulated in mature plants infected with the *∆velA* mutant, while in seedlings inoculated with ∆*velA* and ∆*laeA* mutants, peptide and ammonium transporters were upregulated. This may suggest that in these associations, fungi are under starvation conditions, especially in mature infected plants, as was previously suggested for *sakA*, *noxA*, and *proA* mutant interactions [20]. The observed abnormal in planta hyphal growth of *∆velA* mutant fungi and invasion of vascular bundles are signs of starvation and these hyphae may be acting as a sink to absorb nutrients from host phloem [11]. Our in vitro analyses also showed that ∆*velA* and ∆*laeA* mutants under starvation conditions grow abnormally, but under rich conditions mostly grow normally [11,12]. In vitro analyses of *velA* expression showed the highest levels of its transcripts under starvation conditions [11]. Based on these observations, we speculated that in *E. festucae* during in planta growth, high expression of *velA* suppresses transporters and other starvation response genes so that the fungus stops growing, leading to the growth restriction observed in wild-type associations. Deleting *velA* in *E. festucae* changed its mutualistic interaction with ryegrass to a more antagonistic one and mutant fungi caused death in most inoculated plants. It has been shown that nitrogen metabolism generally plays a key role in pathogenicity [68,69]. Other than nitrogen transporters, no predicted genes involved in nitrogen metabolism were found to be differentially expressed in this study. Changes in nitrogen transport may possibly contribute to host death, with increased fungal growth and insufficient nutrition for the seedlings. This is supported by a comparative transcriptomic study of the *Botrytis cinerea* interaction with *Phaseolus vulgaris* in which it was suggested that upregulation of 2% of DEGs that encode sugar, amino acid, and ammonium transporters and glycoside hydrolases is the reason for the virulence disability of the *∆velA* mutant fungus [34]. It was also suggested that the transcriptomic pattern in this study is a sign of the *∆velA* mutant response to nutrient starvation conditions [34].

In different transcriptomic analyses, VelA regulatory effects on secondary metabolite gene clusters were shown in different fungi [27,28,29,30,31,33,35]. In this study, we showed that VelA positively regulated 48.4% of the gene clusters for secondary metabolism in *E. festucae*, including the two well-known clusters for ergot alkaloids and indole diterpenes. The influence of VelA on ergot alkaloid gene expression in mature plants was much lower than in seedlings inoculated with ∆*velA* and in fact, in mature plants only one gene was differentially expressed, which was upregulated compared with seedlings where most genes were downregulated. VelA regulated indole diterpene gene expression only in infected mature plants, exerting a much weaker influence than seedlings inoculated with the ∆*laeA* mutant. Similar to VelA, NoxA and ProA were shown to have weak regulatory effects on ergot alkaloid and indole diterpene gene expression in infected mature plants, although SakA has a stronger positive regulatory effect [20]. The chemical concentration of ergot alkaloids in mature plants infected with the *∆velA* mutant showed no difference when compared with the wild-type, correlating with the weak regulatory effect of VelA on gene expression. The chemical concentration of most indole diterpenes was significantly greater in ∆*velA* mutant-infected plants, but this did not correlate with greater gene expression. It is likely that the higher biomass of ∆*velA* mutant *E. festucae* in infected plants led to the higher alkaloid levels. There was not always a strong correlation of gene expression with alkaloid production, as also observed in ∆*sakA*, ∆*noxA*, and ∆*proA* mutant associations [20].

Most ergot alkaloids were detected at lower concentrations in ∆*laeA* mutant-infected plants and this was less significant for ∆*velA* mutant-infected plants compared to the wild-type. Despite this, the difference was greater for most of the indole diterpene alkaloids. Therefore, VelA and LaeA exert different influences on alkaloid production in the *E. festucae*–perennial ryegrass association.

Of all the putative SSPs in *E. festucae*, 3.4%, 30%, and 16.7% were differentially expressed in IC ∆*velA*-WT, S ∆*velA*-WT, and IP ∆*velA*-WT, respectively. It seems that VelA is a key regulator of SSP expression in *E. festucae* during its interaction with ryegrass. The greater numbers of differentially expressed SSPs in infected seedlings and mature plants inoculated with the ∆*velA* mutant, compared to in-culture conditions, demonstrated their importance during the infection process and survival. This has also been shown in other pathogenic or symbiotic fungi (reviewed in Presti et al. [59]). Although there are more differentially expressed SSPs in seedlings inoculated with the mutant compared to mature infected plants, a much broader range of fold changes was observed in mature plants. This suggested condition-dependent regulatory roles of VelA for SSPs’ gene expression that possibly are the result of different mechanisms involved during the fungal interaction under these two conditions. VelA, possibly by regulating different SSPs during the infection process and in mature infected plants, positively regulates SSPs that are required for establishing and maintaining symbiotic interaction or negatively regulates SSPs that lead to incompatible interactions. On the other hand, 6.2% and 16% of SSPs were differentially expressed in IC ∆*laeA*-WT and S ∆*laeA*-WT, respectively. Compared to the *velA*-related comparisons, it seems that LaeA has higher regulatory effects in culture and lower in seedlings compared to VelA. This suggested different regulatory effects for these two related global regulators.

In conclusion, this study provides a significant understanding on the strong regulatory effects of VelA on the *E. festucae* transcriptome profile during compatible and incompatible interactions with ryegrass. These regulatory effects could explain some of the observed phenotypes resulting from deleting *velA* in this fungus [11]. It seems that during the interaction of *E. festucae* with ryegrass, VelA controls fungal gene expression to supress or activate genes involved in either inducing or reducing the host plant response.

## 4. Methods

### 4.1. Sample Preparation

Total RNA was extracted from samples comprising fungus grown in culture, perennial ryegrass seedlings, and mature perennial ryegrass plants (Table 1). For culture conditions, *E. festucae* wild-type and ∆*velA* mutant strains were grown for two weeks on cellophane-covered PDA medium in full darkness prior to harvest. Seedlings were grown and harvested as previously described [37]. Endophyte-free seedlings (7–10 d old) of the perennial ryegrass, *L. perenne* ‘Nui’, were inoculated with wild-type and ∆*velA* mutant strains of *E. festucae* (generated previously [11,70]), which were grown for two weeks on PDA medium. Inoculated seedlings were grown for two weeks under 16 h of 650 W/m^2^ light and 8 h of darkness, and after freezing, samples from 4 cm upwards and 0.5 cm downwards from the meristem were collected for RNA extraction and around 100 seedlings for each sample were pooled in three replicates for each treatment.

For the mature plants, the top 4 cm of the newest mature blade of infected plants with different strains were collected in three replicates (three mature plants infected with the same strain) for each treatment.

RNA quality and quantity were determined using an Agilent 2100 Bioanalyzer system (Agilent Technologies, Santa Clara, California, USA), Nanodrop Lite spectrophotometer (Thermo scientific, Waltham, Massachusetts, USA), and gel electrophoresis with 1% agarose gel. RNA samples on dry ice were sent to the Beijing Genomics Institute (BGI, Hong Kong) for sequencing and 2 µg of RNA sample was used to prepare libraries by the BGI standard method (http://www.bgi.com/services/genomics/rna-seqtranscriptome/#tab-id-2). Samples were sequenced in two lanes of an Illumina HiSeq4000 sequencer (paired end, 100-bp reads).

### 4.2. RNA Extraction and Quantitative Real-Time RT-PCR Analysis

RNA extraction and complementary DNA (cDNA) were synthesised from 2 μg of RNA and Real time qPCR was performed with 1 μL of cDNA as described previously [12] using primers that amplified target genes (Table S1). RNA samples alone (no reverse transcription) and water-only (no template) controls were used to detect genomic DNA and experimental contamination. Comparative ΔCt normalized to gamma actin and 60S ribosomal protein L35 was used to calculate transcription levels by using the geNorm algorithm automated in CFX manager software (Bio-Rad, Hercules, California, USA).

### 4.3. HiSeq Results’ Analysis

An analysis of HiSeq results were done as described by Rahnama et al. [37] using a combination RNA-star version 2.5.0c [71] for mapping the HiSeq reads against the genome dataset of *E. festucae* Fl1, with EdgeR package version 3.10.5 [72], for counting the mapped genes. Fold changes and *p*-values were generated using exact tests for differences between two groups of negative binomial counts.

Gene ontology (GO) and functional annotation analysis of differentially expressed genes were performed as described by Rahnama et al. [37].

### 4.4. General Bioinformatics Analyses

Venn diagrams were drawn using the BioVenn online software (http://www.biovenn.nl/) [73]. Volcano plots were drawn in R statistical software environment version 3.2.1 [74].

### 4.5. Availability of Data and Materials

HiSeq Illumina sequencing included 36 raw sequence datasets that have been deposited into the NCBI SRA database with the BioProject ID PRJNA578737.

## Figures and Tables

**Table 1:** Different conditions and comparisons that were used for the transcriptomic analysis.

Conditions	In Culture (IC)	In Seedling (S)	In Planta (IP)
**Host**	PDA medium covered by cellophane	PRG seedlings	Mature infected PRG
**Light condition**	D	8 h L & 16 h D	8 h L & 16 h D
**Time**	14 d	14 dpi	3 months post inoculation
***E. festucae* strains Comparisons**	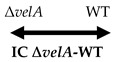	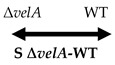	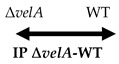

WT, wild-type; D, dark; L, light; PRG, perennial ryegrass.

**Table 2:** DEGs with transporter activity in all different comparisons.

		Fold Change					
Model	Predicted Function	IC ∆*laeA*-WT	S ∆*laeA*-WT	IC ∆*velA*-WT	S ∆*velA*-WT	IP ∆*velA*-WT	Presence in Core Set
EfM3.003420	Phospholipid transporter	−1.1	−1.2	−1.1	−1.1	2.5	No
EfM3.007320	Copper transporter	1.4	1.6	1.2	2.8	1.9	No
EfM3.009680	ABC transporter	−1.9	−1.8	−1.9	−1.7	1.2	No
EfM3.009730	ABC transporter	−2.0	-2.2	−1.7	−1.8	−2.1	No
EfM3.012390	Nitrate transporter	1.6	1.0	1.3	1.0	4.9	No
EfM3.012760	Malic acid transporter	1.5	−1.7	−1.0	−3.0	−1.4	Yes
EfM3.014790	ABC transporter	1.2	−1.8	−2.1	−1.9	−1.4	No
EfM3.018210	Proline-specific permease	−1.1	1.1	−1.0	1.2	−2.2	No
EfM3.020140	Sulphate transporter	1.1	1.4	1.2	2.5	−1.7	No
EfM3.025050	Amino acid permease	1.4	1.3	1.1	2.2	1.1	No
EfM3.025350	Transporter	−1.3	−1.3	−1.7	1.2	−1.1	No
EfM3.027520	ABC transporter	1.1	1.7	1.2	3.1	2.8	No
EfM3.027540	ABC transporter	1.3	1.4	1.4	2.5	2.8	No
EfM3.027570	Peptide transporter	2.6	3.1	3.0	9.9	12.5	Yes
EfM3.032550	ABC transporter	1.2	1.3	1.1	−1.2	1.2	No
EfM3.035410	Carboxylic acid transporter	1.4	2.0	−1.1	−1.0	5.1	Yes
EfM3.039020	Ammonium transporter	1.0	2.0	1.0	3.0	4.5	No
EfM3.040210	Phospholipid-translocating ATPase	−1.2	−1.1	−1.1	−1.1	2.4	No
EfM3.045520	Hydrogen ion transmembrane transporter	3.4	−2.4	8.6	−8.4	−4.0	No
EfM3.047210	Purine permease	−1.1	1.2	−1.1	1.3	2.1	No
EfM3.055090	ABC multidrug transporter	1.1	−1.2	−1.1	−2.7	1.3	No
EfM3.056220	ABC multidrug transporter	1.2	−4.8	−1.1	−4.2	−3.4	Yes
EfM3.058970	Na+/H+ antiporter	1.0	−1.1	−1.1	−1.1	−2.1	No
EfM3.066900	Xanthine/uracil permease	1.1	−1.6	1.4	−2.9	-6.1	Yes
EfM3.074200	Transmembrane transporter	2.0	−1.3	2.8	−2.6	−1.6	No
EfM3.075680	Transmembrane transporter	−1.8	−2.6	−2.1	−2.4	−2.4	No
EfM3.000930	Zinc ion transporter	1.1	−2.8	−1.1	−1.4	1.1	No
EfM3.005950	Amino acid permease	1.4	2.4	−1.0	1.5	1.4	No
EfM3.029800	ABC multidrug transporter	2.4	1.1	1.1	−1.5	−1.1	No
EfM3.064820	Sugar transporter	−1.7	−2.4	−1.2	1.2	−1.2	No
EfM3.077030	Inositol transporter	1.3	2.2	1.2	1.4	1.1	No
EfM3.080140	Calcium transporter	−1.1	−2.4	−1.0	−1.6	1.8	No
EfM3.158840	Metal ion transporter	1.1	2.0	1.0	2.0	1.1	No

**Table 3:** DEGs with host cell wall degradation activity. Fold changes showed in bold are statistically significant (FDR ≤ 0.05).

				Fold Change				
Model	CAZyme Class	Predicted Function	Target Cell Wall Component	IC ∆*laeA*-WT	S ∆*laeA*-WT	IC ∆*velA*-WT	S ∆*velA*-WT	IP ∆*velA*-WT
EfM3.049570	GH13	Alpha-glucosidase	Cellulose	1.0	−2.1	−1.2	−1.5	−1.2
EfM3.053990	GH5	Endoglucanase	Cellulose	1.5	2.1	−1.1	1.1	1.3
EfM3.005420	N/A	Exo-1,4-beta-xylosidase	Hemicellulose	1.1	1.9	−1.0	2.3	1.5
EfM3.040190	GH10	Endo-1,4-beta-xylanase	Hemicellulose	1.7	3.3	1.1	3.9	83.5
EfM3.037040	GH62	Glycosyl hydrolase	Hemicellulose	1.1	4.3	1.0	4.3	69.6
EfM3.008730	CE8	Pectin methylesterase	Pectin	−1.3	1.7	1.0	2.0	1.4
EfM3.008610	CE5	Cutinase	Cutin	2.5	2.9	1.2	−2.0	2.9
EfM3.005300	N/A	Cuticle-degrading protease	Cuticle	1.0	−2.8	1.0	−5.1	−4.1

**Table 4:** Differentially expressed genes engage in fungal cell wall composition. Fold changes showed in bold are statistically significant (FDR ≤ 0.05).

			Fold Change				
Model	CAZyme Class	Predicted Function	IC ∆*laeA*-WT	S ∆*laeA*-WT	IC ∆*velA*-WT	S ∆*velA*-WT	IP ∆*velA*-WT
EfM3.000810	CBM18	Chitinase	1.0	−3.0	1.0	−3.3	−3.1
EfM3.024310	GH18	Endochitinase B1	1.4	1.6	−1.1	1.9	2.1
EfM3.049120	GT2	Chitin synthase	−1.3	−1.4	2.0	1.3	−1.4
EfM3.056450	CBM43	Glucanosyl transferase	−1.2	−1.2	−1.0	−1.2	2.1
EfM3.056810	GH64	Glucan endo-1,3-beta-glucosidase	1.1	1.1	−1.1	1.0	−2.2
EfM3.078790	N/A	Cell wall protein SED1	1.1	−3.4	1.1	−2.5	−1.8
EfM3.054000	N/A	Related to cell wall glycoprotein	−1.7	1.1	1.1	2.3	1.9
EfM3.034340	N/A	Cell wall glycoprotein	−1.2	−3.4	1.4	−1.9	−3.7

## Supplementary Materials

Supplementary materials can be found at https://www.mdpi.com/2076-2607/8/1/33/s1.
