# Supplementary material for: VelA and LaeA are Key Regulators of Epichloë festucae Transcriptomic Response during Symbiosis with Perennial Ryegrass
Source: Microorganisms. 2019 Dec 23;8(1):33. doi: 10.3390/microorganisms8010033 (PMC7023048; doi:10.3390/microorganisms8010033)
Supplement: Supplementary file 1 [file microorganisms-08-00033-s001.pdf]

Table S1. Primers for qRT-PCR used in this study.

Name	Sequence (5'-3')	Purpose	Gene Model
LaeAq-F	GGTACCGGAATATGGGCCATT	*laeA* Fwd	EfM3.069170
LaeAq-R	GAATGAGGGCTGGCTGAATC	*laeA* Rev	
VelAq2-F	GCTCAGGGATGAAGGCGAAT	*velA* Fwd	EfM3.049680
VelAq3-R	TGGCGTTGTAGTCGAAAGTGA	*velA* Rev	
Actinq.IDT-F	GTCTTGAGTCTGGCGGTATC	*gamma actin* Fwd	EfM3.017740
Actinq.IDT-R	CTTCTGCATACGGTCGGAG	*gamma actin* Rev	
60SqIDT-F	GCAGCAAGTTGAACAAGATCC	60S ribosomal protein L35	EfM3.073270
60SqIDT-R	CACGGGTTTGCTTGACAC	60S ribosomal protein L35	
perA-F	TGACGGTTGAAGTATGGCTG	*perA* Fwd	EfM3.018710
perA-R	TCTGTGAAGGTGTGGCATG	*perA* Rev	
ltmG-F	TGACCGACGCCATTAATGAG	*ltmG* Fwd	EfM3.182880
ltmG-R	TCCACGTCGCAGTTTAGATG	*ltmG* Rev	
dmaWq-F	CCACTCGTACATTTCCTTCTC	*dmaW* Fwd	EfM3.065770
dmaWq-R	TTCGGGAAACACAGGCCATTC	*dmaW* Rev	

Table S2- General description of mRNA-sequencing results

mRNA-sequencing reads	In culture	In seedling	In planta
Total read number	1,132,721,194	905,625,652	810,694,678
Number of total reads after quality trimming	1,131,271,924	904,675,122	809,938,396
Percentage of total reads after quality trimming	99.87%	99.90%	99.91%
Number of mapped reads	1,083,417,500	764,871,314	724,293,326
Proportion of mapped reads (% of trimmed reads)	95.77%	84.55%	89.43%
Number of reads mapped to Endophyte genome	1,080,853,724	48,000,483	13,360,773
Percentage of mapped fungal reads to total mapped reads	99.76%	6.28%	1.84%

Table S3- Percentage of DEGs with their primary functions onto 'Biological Process' Gene Ontology.

Model	Biological process categories	Percentage of DEGs		
		**IC Δ*velA*-WT**	**S Δ*velA*-WT**	**IP Δ*velA*-WT**
GO:0032502	**developmental process**	0.00	0.00	0.44
GO:0023052	**signaling**	1.75	1.71	1.33
GO:0050789	**regulation of biological process**	8.77	4.44	5.33
GO:0000003	**reproduction**	1.75	0.34	0.44
GO:0016043	**cellular component organization**	0.00	0.34	3.11
GO:0071554	**cell wall organization or biogenesis**	1.75	1.02	1.33
GO:0065007	**biological regulation**	10.53	5.12	6.67
GO:0048518	**positive regulation of biological process**	1.75	1.02	1.78
GO:0048519	**negative regulation of biological process**	3.51	0.68	0.89
GO:0019740	**nitrogen utilization**	1.75	0.68	0.44
GO:0051704	**multi-organism process**	1.75	1.71	1.78
GO:0009987	**cellular process**	28.07	18.43	25.33
GO:0022414	**reproductive process**	1.75	0.34	0.44
GO:0023046	**signaling process**	1.75	1.37	0.89
GO:0008152	**metabolic process**	33.33	38.91	43.11
GO:0051234	**establishment of localization**	8.77	6.48	9.78
GO:0051179	**localization**	10.53	6.83	10.22
GO:0040007	**growth**	0.00	0.34	0.44
GO:0050896	**response to stimulus**	1.75	3.07	3.11
GO:0044085	**cellular component biogenesis**	1.75	0.68	1.33
	**No GO**	68.42	57.00	61.33

Table S4- Differential expression of different velvet component in different comparisons. Fold changes show in bold are statistically significant (FDR≤0.05) changed more than two times.

	**IC Δ*laeA*-WT**	**S Δ*laeA*-WT**	**IC Δ*velA*-WT**	**S Δ*velA*-WT**	**IP Δ*velA*-WT**
velD	1.12	2.00	-1.03	2.24	-1.36
velC	1.40	1.58	1.42	-1.19	2.55
velB	-1.14	1.17	-1.11	1.33	-1.05
velA	1.01	1.03	**-3.20**	**-3.46**	**-4.69**
laeA	**-3.50**	**-2.13**	**2.22**	**2.48**	1.07

Table S5- Homologues of known fungal plant cell wall degrading enzymes in *E. festucae*

Model	Family	E-value	Category	Gene Family
EfM3.016930	1	3.00E-23	Accessary - cellulose	Alpha-glucosidase (Type 1)
EfM3.024120	1	0	Accessary - cellulose	Alpha-glucosidase (Type 1)
EfM3.050100	1	1.00E-25	Accessary - cellulose	Alpha-glucosidase (Type 1)
EfM3.022450	2	0	Accessary - cellulose	Alpha-glucosidase (Type 2)
EfM3.049570	2	5.00E-17	Accessary - cellulose	Alpha-glucosidase (Type 2)
EfM3.010800	3	0	Accessary - pectin	Beta-D-galactosidase (Type 1)
EfM3.010800	3	0	Accessary - pectin	Beta-D-galactosidase (Type 1)
EfM3.053570	3	3.00E-20	Accessary - pectin	Beta-D-galactosidase (Type 1)
EfM3.075210	4	0	Accessary - pectin	Beta-D-galactosidase (Type 2)
EfM3.008730	6	5.00E-106	Accessary - pectin	Pectin methylesterase
EfM3.008610	9	7.00E-81	Leaf Surface - Cutin	Cutinase
EfM3.066110	9	4.00E-60	Leaf Surface - Cutin	Cutinase
EfM3.030520	10	2.00E-12	Main-chain degrading - cellulose	Cellobiohydrolase (Type 1)
EfM3.032730	12	0	Main-chain degrading - glacto(gluco)mannan	Alpha-mannosidase (Type 1)
EfM3.039340	12	3.00E-63	Main-chain degrading - glacto(gluco)mannan	Alpha-mannosidase (Type 1)
EfM3.042900	12	0	Main-chain degrading - glacto(gluco)mannan	Alpha-mannosidase (Type 1)
EfM3.044230	12	0	Main-chain degrading - glacto(gluco)mannan	Alpha-mannosidase (Type 1)
EfM3.073730	12	0	Main-chain degrading - glacto(gluco)mannan	Alpha-mannosidase (Type 1)
EfM3.078610	12	0	Main-chain degrading - glacto(gluco)mannan	Alpha-mannosidase (Type 1)
EfM3.079010	12	2.00E-29	Main-chain degrading - glacto(gluco)mannan	Alpha-mannosidase (Type 1)
EfM3.015680	13	0	Main-chain degrading - glacto(gluco)mannan	Alpha-mannosidase (Type 2)
EfM3.020350	15	0	Main-chain degrading - glacto(gluco)mannan	Beta-mannosidase
EfM3.056680	19	2.00E-27	Main-chain degrading - pectin	Polygalacturonase
EfM3.061190	19	6.00E-85	Main-chain degrading - pectin	Polygalacturonase
EfM3.040190	21	6.00E-98	Main-chain degrading - xylan	Endoxylanase (Type 1)
EfM3.050060	22	1.00E-62	Main-chain degrading - xylan	Endoxylanase (Type 2)

Table S6- Differentially expressed genes of secondary metabolism gene clusters in different comparisons. Fold changes show in bold are statistically significant (FDR≤0.05) changed more than two times. Empty fold change cells are representative of not expressed gene in both Δ*laeA* and wild type strains. Clusters highlighted in grey colour are including DEGs. Alk: alkaloids, K: polyketide synthase, N: nonribosomal peptide synthetase, D: DMATS-family prenyltransferase, T: terpene cyclase, Misc.: miscellaneous.

			**Fold change**				
	**Cluster**	**Cluster Type[a]**	**IC Δ*laeA*-WT**	**S Δ*laeA*-WT**	**IC Δ*velA*-WT**	**S Δ*velA*-WT**	**IP Δ*velA*-WT**
EfM3.049620	1	Alk, D, N	**9.2**	**-10.3**	**9.7**	**-13.4**	**4.1**
EfM3.049630	1	Alk, D, N	**2.1**	**-4.8**	-1.1	**-4.2**	4.0
EfM3.049640	1	Alk, D, N	1.8	-7.3	1.9	**-14.5**	2.4
EfM3.049650	1	Alk, D, N	-1.2	**-8.4**	2.3	**-4.6**	2.7
EfM3.049660	1	Alk, D, N	1.2	**-5.6**	1.4	**-9.7**	-1.0
EfM3.049670	1	Alk, D, N	-1.2	**-5.0**	-1.3	**-4.1**	2.6
EfM3.063200	1	Alk, D, N	-1.0	**-2.7**	-1.1	**-2.3**	1.5
EfM3.065750	1	Alk, D, N	1.5	**-4.8**	1.3	**-2.5**	1.1
EfM3.065755	1	Alk, D, N	1.2	**-5.3**	1.0	**-4.1**	-1.0
EfM3.065760	1	Alk, D, N	-7.0	**-7.8**	-1.9	**-2.3**	2.3
EfM3.065770	1	Alk, D, N	3.4	**-34.7**	3.5	**-5.0**	2.0
EfM3.048150	2	Alk, T, D	-1.7	**-2.5**	1.7	-1.5	-1.8
EfM3.048160	2	Alk, T, D	1.2	**-2.7**	1.3	-1.8	-1.7
EfM3.048170	2	Alk, T, D	1.3	**-2.0**	1.1	-1.8	**-2.0**
EfM3.048180	2	Alk, T, D	-1.6	**-4.4**	-1.5	-1.5	-1.6
EfM3.048190	2	Alk, T, D	1.0	**-2.5**	-1.2	-1.7	**-2.1**
EfM3.048210	2	Alk, T, D	1.1	-1.6	1.1	-1.4	-1.5
EfM3.182620	2	Alk, T, D	1.7	**-4.8**	1.2	-1.8	**-2.7**
EfM3.182630	2	Alk, T, D	-1.0	**-3.9**	-1.3	-1.5	**-3.1**
EfM3.182880	2	Alk, T, D	1.4	**-2.8**	1.2	-1.3	-1.7
EfM3.182890	2	Alk, T, D	1.2	-1.2	-1.4	-1.6	-1.9
EfM3.018710	4	Alk, N	1.0	**-1.9**	1.1	-1.3	-1.4
EfM3.003180	5	K	-1.1	-1.2	1.0	-1.0	-3.2
EfM3.003190	5	K	1.0	-1.0	-1.1	1.2	1.1
EfM3.003200	5	K	1.2	1.8	-1.0	1.6	-1.2
EfM3.003210	5	K	0.0	0.0	1.0	0.0	0.0
EfM3.075070	6	K	1.0	1.1	1.0	-1.0	-1.4
EfM3.075080	6	K	1.1	1.1	1.1	1.0	1.1
EfM3.075090	6	K	1.1	-1.2	1.0	1.2	1.4
EfM3.075100	6	K	1.2	1.1	1.0	1.3	-2.3

EfM3.075110	6	K	1.1	-1.2	-1.0	-1.5	-1.0
EfM3.005360	7	K	1.1	-1.5	-1.1	-1.7	-1.1
EfM3.005370	7	K	-1.1	-1.2	1.0	-1.5	1.6
EfM3.005380	7	K	-1.5	1.0	-1.1	1.1	-1.2
EfM3.005390	7	K	1.0	-1.2	-1.0	1.1	-1.7
EfM3.005400	7	K	1.4	2.2	1.3	1.7	**10.1**
EfM3.005410	7	K	1.7	**3.8**	1.2	3.5	3.2
EfM3.005420	7	K	1.1	1.9	-1.0	**2.3**	1.5
EfM3.005430	7	K	-1.1	1.2	-1.1	-1.0	1.1
EfM3.005440	7	K	-1.5	**-5.4**	1.0	**-2.8**	-1.3
EfM3.005450	7	K	-1.4	**-6.9**	-1.1	**-2.5**	1.1
EfM3.005460	7	K	-1.3	**-2.1**	-1.2	-1.9	1.7
EfM3.005470	7	K	-1.0	-1.2	1.0	-1.3	1.3
EfM3.005480	7	K	-1.1	1.0	-1.1	1.2	1.4
EfM3.109450	7	K					
EfM3.042030	8	K	-1.0	1.8	2.0	1.5	-1.3
EfM3.042040	8	K	-1.5	-1.1	-1.1	-5.8	0.0
EfM3.042050	8	K	1.3	0.0	1.2	0.0	0.0
EfM3.042060	8	K					
EfM3.042070	8	K	1.4	-10.0	-2.7	-2.3	0.0
EfM3.042080	8	K	-1.1	1.1	-1.4	-1.1	2.1
EfM3.042090	8	K	-1.0	1.0	-1.3	-1.5	1.0
EfM3.042100	8	K	-1.1	1.0	-1.2	1.2	-1.2
EfM3.042110	8	K	1.8	-1.1	1.3	-1.6	1.2
EfM3.042120	8	K	0.0	0.0	1.0	0.0	0.0
EfM3.042130	8	K	1.5	1.1	1.3	-1.1	2.0
EfM3.105180	8	K					
EfM3.047050	10	K	-1.0	-1.1	-1.0	1.1	-1.0
EfM3.047060	10	K	-1.0	-1.1	1.0	1.0	-1.0
EfM3.047070	10	K	**-3.0**	**-4.1**	-1.3	-1.7	-1.2
EfM3.047080	10	K	-1.6	1.6	1.2	2.8	1.7
EfM3.048220	11	K	1.1	-1.1	1.0	-1.2	-1.9
EfM3.048230	11	K	1.5	0.0	2.5	4.0	3.0
EfM3.048240	11	K	1.1	-1.2	-1.1	-1.3	-1.6
EfM3.048260	11	K	1.4	-1.9	-1.4	2.3	**-8.9**
EfM3.048270	11	K	-1.4	3.3	1.8	-2.0	0.0
EfM3.048280	11	K	-1.2	-1.1	1.1	1.4	-1.3
EfM3.182920	11	K					
EfM3.182950	11	K	1.2	-1.7	-1.7	**-14.0**	0.0
EfM3.057450	13	K	1.1	1.0	-1.0	1.1	-1.0
EfM3.057460	13	K	-1.1	-1.3	-1.4	1.3	1.2
EfM3.057470	13	K	1.3	-1.1	-1.5	-1.9	1.2
EfM3.062090	13	K	1.3	1.0	1.0	-1.4	1.3

EfM3.062100	13	K	-1.2	-1.7	-1.4	-1.8	-1.5
EfM3.062110	13	K	-1.1	-1.2	-1.2	1.2	-1.0
EfM3.037150	20	K-N	-1.3	-1.2	-1.0	-1.3	-1.1
EfM3.037160	20	K-N	-1.0	1.7	1.1	**2.1**	-1.0
EfM3.037170	20	K-N	1.1	-1.0	1.1	-1.1	1.2
EfM3.037180	20	K-N	1.0	-1.2	-1.0	-1.9	1.6
EfM3.037190	20	K-N	-1.1	-1.3	1.0	-1.2	-1.4
EfM3.037200	20	K-N	-1.1	-1.1	1.1	-1.0	-1.1
EfM3.037210	20	K-N	-1.0	1.6	1.0	1.2	-1.5
EfM3.037220	20	K-N	-1.0	1.3	1.1	1.5	-1.1
EfM3.037230	20	K-N	-1.0	-1.1	-1.0	-1.3	1.4
EfM3.037240	20	K-N	-1.1	1.0	1.0	-1.2	1.2
EfM3.037250	20	K-N	-1.1	-1.1	-1.0	1.2	-1.1
EfM3.037260	20	K-N	1.0	-1.1	-1.2	-1.1	-1.1
EfM3.037270	20	K-N	0.0	0.0	1.0	0.0	0.0
EfM3.037280	20	K-N	1.6	-1.3	1.0	-1.1	-2.0
EfM3.037290	20	K-N	-1.0	-1.2	-1.0	-1.2	1.2
EfM3.038470	21	K, D	1.1	1.3	1.1	-1.5	-3.0
EfM3.038480	21	K, D	0.0	0.0	1.0	0.0	0.0
EfM3.038490	21	K, D	1.0	-1.2	-1.0	-1.3	1.2
EfM3.038500	21	K, D	-1.0	1.4	-1.1	1.8	1.3
EfM3.038505.<br>pseudo	21	K, D	0.0	0.0	1.0	0.0	0.0
EfM3.038510	21	K, D	-1.0	1.4	1.0	1.5	1.1
EfM3.038520	21	K, D	1.0	1.0	1.0	1.1	-1.1
EfM3.038530	21	K, D	1.4	-1.0	1.1	-1.1	-1.9
EfM3.038540	21	K, D	1.2	-2.1	-1.6	-1.6	1.0
EfM3.038550	21	K, D	1.1	-3.0	-1.2	1.1	1.0
EfM3.038560	21	K, D	-1.1	-1.6	-1.0	-2.5	1.3
EfM3.038570	21	K, D	-1.1	-6.0	-1.2	-4.8	-1.7
EfM3.038580	21	K, D	1.0	-1.1	1.1	-1.0	-1.2
EfM3.038590	21	K, D	1.1	1.1	1.0	1.0	-1.2
EfM3.038600	21	K, D	-1.1	-1.0	1.2	-1.1	1.4
EfM3.014730	22	K, K	-1.2	1.3	-1.2	-2.0	1.4
EfM3.014750	22	K, K	**4.2**	1.6	-2.6	1.3	-1.2
EfM3.014760	22	K, K	1.0	**-2.1**	-1.5	**-2.1**	-1.7
EfM3.014770	22	K, K	-1.0	-2.1	-1.6	-2.0	**-2.1**
EfM3.014780	22	K, K	1.4	**-4.4**	1.2	**-5.5**	**-8.5**
EfM3.014790	22	K, K	1.2	-1.8	**-2.1**	-1.9	-1.4
EfM3.014800	22	K, K	**2.4**	-1.9	1.6	-1.7	-1.4
EfM3.014810	22	K, K	1.1	1.1	**-2.1**	-1.1	-1.4
EfM3.014820	22	K, K	1.7	-1.4	-1.4	-1.3	-1.5
EfM3.014830	22	K, K	1.2	**-2.1**	-1.1	-1.4	-1.2

EfM3.185360	22	K, K	**4.7**	-1.3	2.6	-1.2	**-2.1**
EfM3.185370	22	K, K					
EfM3.048420	24	N	1.5	1.3	1.0	-1.1	-1.1
EfM3.048430	24	N	-1.0	-1.4	-1.0	-1.1	1.0
EfM3.009640	25	N	-1.2	-1.6	-1.2	1.1	-1.0
EfM3.009650	25	N	**-4.0**	**-3.5**	**-2.4**	**-2.2**	-2.0
EfM3.009660	25	N	**-2.6**	**-2.7**	**-2.2**	**-2.0**	-1.1
EfM3.009670	25	N	**-2.0**	**-2.2**	-1.9	-1.8	-1.1
EfM3.009680	25	N	-1.9	-1.8	-1.9	-1.7	1.2
EfM3.009690	25	N	-1.4	-1.6	-1.2	-1.6	-1.4
EfM3.009700	25	N	-1.4	**-2.1**	-1.5	**-2.2**	-1.8
EfM3.009710	25	N	**-2.7**	**-2.5**	**-2.3**	-1.9	1.6
EfM3.009720	25	N	**-3.1**	**-2.6**	**-2.3**	**-2.4**	-7.7
EfM3.009730	25	N	-2.0	**-2.2**	-1.7	-1.8	**-2.1**
EfM3.110710	25	N	-1.1	-2.2	-1.2	1.3	-11.8
EfM3.037640	26	N	1.0	-1.1	1.1	-1.1	-1.1
EfM3.050020	27	N	-1.2	-1.1	1.0	-1.1	1.2
EfM3.176040	27	N					
EfM3.176050	27	N	-1.2	-1.1	1.1	1.4	1.2
EfM3.053270. pseudo	28	N	3.2	-6.8	4.9	-6.2	0.0
EfM3.053280	28	N	-1.8	2.5	1.0	2.1	0.0
EfM3.053290	28	N	-1.1	**-5.9**	-1.3	**-2.9**	-3.0
EfM3.053300	28	N	1.1	**-6.9**	1.8	**-4.5**	**-7.5**
EfM3.075660	29	N	-1.4	-2.1	-1.2	-1.6	-1.7
EfM3.075670	29	N	-1.8	-3.2	-1.8	-1.5	-2.1
EfM3.075680	29	N	-1.8	-2.6	**-2.1**	-2.4	-2.4
EfM3.075690	29	N	1.1	-1.2	1.0	1.0	-1.5
EfM3.075700	29	N	1.1	1.2	-1.1	1.2	-1.0
EfM3.145530	29	N	0.0	0.0	1.0	0.0	0.0
EfM3.082140	31	N	-1.0	-1.1	-1.0	1.2	-1.1
EfM3.106500	31	N	1.1	1.3	-1.1	1.1	1.1
EfM3.106510	32	N	1.6	-1.8	-1.2	-5.2	-1.5
EfM3.106520	32	N					
EfM3.106530	32	N					
EfM3.106540	32	N	-3.3	3.7	-1.3	0.0	0.0
EfM3.106550	32	N					
EfM3.106560	32	N					
EfM3.005240	33	N	-1.0	1.1	-1.1	1.2	-1.1
EfM3.005350	33	N	1.0	-1.5	-1.0	-1.2	1.9
EfM3.081180	33	N					
EfM3.019590	34	N	1.4	-1.3	1.8	1.9	17.9
EfM3.029750	35	N	-1.0	-1.4	-1.0	-1.3	-1.0

EfM3.029760	35	N	-1.1	-1.1	1.0	-1.0	1.1
EfM3.029770	35	N	1.4	1.3	1.2	-1.4	1.1
EfM3.029780	35	N	1.1	-1.1	1.0	1.0	-1.1
EfM3.029790	35	N	**3.3**	1.1	1.4	-1.1	-1.3
EfM3.029800	35	N	**2.4**	1.1	1.1	-1.5	-1.1
EfM3.053820	37	N	1.2	-1.2	1.0	1.2	1.3
EfM3.053830	37	N	1.2	1.5	-1.1	-1.3	-1.1
EfM3.053840	37	N	1.2	1.1	-1.0	-1.1	-1.1
EfM3.054970	38	N	-1.1	1.2	-1.1	-1.0	1.2
EfM3.055060	38	N	2.5	1.6	2.5	-3.5	0.0
EfM3.055070	38	N	1.0	-1.0	-1.1	1.1	1.8
EfM3.055080	38	N	-2.0	-3.9	1.3	**-58.0**	**-55.3**
EfM3.055090	38	N	1.1	-1.2	-1.1	**-2.7**	1.3
EfM3.055100	38	N	-1.1	-1.2	-1.1	-1.2	-1.1
EfM3.055110. pseudo	38	N	-1.0	1.1	1.0	-1.0	-1.0
EfM3.056220	39	N	1.2	**-4.8**	-1.1	**-4.2**	**-3.4**
EfM3.056230	39	N	1.3	**-8.4**	-1.3	**-10.7**	**-3.8**
EfM3.056240	39	N	1.3	-1.7	-1.0	-2.1	**-4.7**
EfM3.062310	39	N	-2.8	**-7.5**	-1.7	**-18.5**	**-7.5**
EfM3.062320	39	N	-5.4	-7.4	-8.2	-6.0	**-4.2**
EfM3.062330	39	N	-1.0	-1.4	-1.0	-1.2	**-3.0**
EfM3.062340	39	N	1.0	-1.4	1.0	-1.3	**-3.2**
EfM3.036920	42	N	-1.2	-1.2	-1.1	-1.0	1.1
EfM3.014880	44	N, D	1.2	1.7	-1.1	-3.5	-1.7
EfM3.014890	44	N, D	-1.2	-1.4	-1.7	-20.7	-3.5
EfM3.014900	44	N, D	-1.1	-5.5	-1.4	-2.4	1.3
EfM3.014910	44	N, D	1.3	-2.5	1.0	-1.4	2.0
EfM3.014920	44	N, D	-1.6	**-3.2**	-1.6	**-6.9**	8.7
EfM3.014930	44	N, D	-1.9	3.6	-23.3	4.0	0.0
EfM3.014940	44	N, D	-1.2	-6.8	1.6	-6.2	0.0
EfM3.014950	44	N, D	1.1	1.2	-1.4	-1.5	5.1
EfM3.014960	44	N, D	1.0	-13.2	1.3	-12.0	12.9
EfM3.014970	44	N, D	-1.0	-1.4	-1.3	-1.4	-1.5
EfM3.049580	45	T	-1.6	**-2.4**	-1.3	**-2.8**	**-2.4**
EfM3.049590	45	T	1.1	-1.8	-1.1	-1.8	-2.1
EfM3.053860	45	T	-1.3	-1.5	-1.3	-2.0	-1.3
EfM3.053870	45	T	-1.1	**-2.1**	-1.2	-1.7	**-2.3**
EfM3.053880	45	T	1.0	-1.3	-1.0	-1.3	-1.1
EfM3.063000	45	T	-1.2	1.3	-1.1	-1.5	-1.9
EfM3.071710	45	T	1.0	-1.5	1.0	-1.9	-2.7
EfM3.112070	45	T					
EfM3.122680	45	T	-1.1	-1.6	-1.1	-1.5	-1.2

EfM3.010850	49	Misc.	1.2	1.1	-1.1	-1.3	-1.9
EfM3.010860	49	Misc.	-1.0	1.1	-1.0	-1.3	-1.6
EfM3.010870	49	Misc.	-1.1	-1.3	-1.0	-1.2	-2.0
EfM3.010880	49	Misc.	1.1	-1.1	1.0	-1.2	-1.5
EfM3.054860	50	Misc.	1.1	1.1	1.1	-1.7	-1.1
EfM3.054870	50	Misc.	1.0	**-2.9**	-1.2	-2.1	1.1
EfM3.054880	50	Misc.	1.7	-2.9	-1.1	1.2	1.5
EfM3.054890	50	Misc.	1.2	2.0	-1.0	2.1	12.9
EfM3.054900	50	Misc.	1.4	9.3	1.5	9.8	4.9
EfM3.054910	50	Misc.	-9.1	0.0	-4.7	0.0	0.0
EfM3.054930	50	Misc.	1.2	1.5	-1.0	-1.0	6.2
EfM3.054940	50	Misc.	-1.0	1.0	1.0	-1.2	-1.2
EfM3.173100	50	Misc.					

Table S7- Putative small secreted proteins that differentially expressed in at least one of comparisons. Bold numbers are the fold changes that are significantly (FDR ≤ 0.05) changed more than two times.

Gene	Name	IC Δ*laeA*-WT	S Δ*laeA*-WT	IC Δ*velA*-WT	S Δ*velA*-WT	IP Δ*velA*-WT	Presence in core set	Blast accession number	blast functions
EfM3.001305	-	**-2.2**	-3.5	-1.5	**-4.1**	-5.16	No	KID97701	Killer toxin, Kp4, partial [Metarhizium majus ARSEF 297]
EfM3.001310	-	-1.6	-1.4	-1.3	**-2.7**	-1.54	No	XP_014544272	Calcium Channel Inhibitor, partial [Metarhizium brunneum ARSEF 3297]
EfM3.005070	-	-1.2	1.3	-1.2	**2.4**	**3.11**	No	XP_018136637	cupin superfamily protein [Pochonia chlamydosporia 170]
EfM3.006460	-	1.3	**-8.4**	1.1	**-20.3**	**-7.06**	No	KZZ96531	hypothetical protein AAL_03760 [Moelleriella libera RCEF 2490]
EfM3.007440	-	1.2	-1.5	-1.4	**-2.3**	-1.32	No	XP_018140327	hypothetical protein VFPPC_11200 [Pochonia chlamydosporia 170]
EfM3.007580	-	1.3	**3.6**	1.0	**3.1**	**15.08**	No	KFG80511	hypothetical protein MANI_022663 [Metarhizium anisopliae]
EfM3.007740	-	1.7	1.1	**2.7**	**-3.0**	2.28	No	CCE34646	uncharacterized protein CPUR_08580 [Claviceps purpurea 20.1]
EfM3.008680	-	1.8	1.4	1.2	**2.2**	1.23	Yes	NA	
EfM3.008740	-	**-2.8**	1.7	-1.1	**2.7**	**4.04**	Yes	NA	
EfM3.009460	-	1.3	1.2	-1.0	-1.1	**6.46**	No	XP_018140951	hypothetical protein VFPPC_09225 [Pochonia chlamydosporia 170]
EfM3.014350	sspO	1.1	**-2.5**	-1.0	**-5.1**	**-2.72**	Yes	KJZ74698	hypothetical protein HIM_05815 [Hirsutella minnesotensis 3608]
EfM3.016770	sspM	1.6	**-2.9**	1.3	**-29.3**	**-31.24**	Yes	ELA34989	hypothetical protein CGGC5_5250 [Colletotrichum fructicola Nara gc5]
EfM3.026630	-	**2.3**	**6.2**	1.1	**4.5**	**72.39**	Yes	NA	
EfM3.027550	-	**2.3**	**2.4**	**2.0**	**3.7**	1.75	No	NA	
EfM3.033250	-	1.0	**6.0**	-1.1	**4.0**	2.94	No	PHH60641	hypothetical protein CDD81_1392 [Ophiocordyceps australis]
EfM3.033650	-	1.1	1.0	1.0	1.1	**3.09**	No	NA	
EfM3.034400	-	1.1	1.1	-1.2	**4.1**	5.58	No	NA	
EfM3.038640	-	-1.2	-1.2	1.0	**-2.3**	-1.68	No	XP_007825160	hypothetical protein MAA_08971 [Metarhizium robertsii ARSEF 23]
EfM3.040200	-	-1.9	-1.2	-1.1	1.3	**41.56**	Yes	XP_007825947	hypothetical protein MAA_09758 [Metarhizium robertsii ARSEF 23]
EfM3.041600	-	1.2	1.8	-1.1	**2.2**	**16.36**	No	XP_018139722	hypothetical protein VFPPC_14197 [Pochonia chlamydosporia 170]
EfM3.041760	-	1.4	**2.0**	-1.0	1.3	2.42	No	NA	
EfM3.041770	-	1.1	1.8	1.0	**4.7**	**55.32**	Yes	NA	
EfM3.042450	-	-1.7	**-2.2**	-1.5	**-2.1**	-1.02	No	PVI00678	hypothetical protein DM02DRAFT_614138 [Periconia macrospinosa]
EfM3.042700	-	-1.9	**3.0**	2.0	**2.6**	**2.76**	No	CCE27014	uncharacterized protein CPUR_00486 [Claviceps purpurea 20.1]
EfM3.043290	-	1.2	1.2	-1.1	**2.4**	1.36	No	XP_018176067	hypothetical protein VFPFJ_07599 [Purpureocillium lilacinum]
EfM3.045040	-	1.1	**-2.9**	-1.0	-1.1	-2.12	No	NA	

EfM3.048790	-	**2.6**	1.8	1.5	1.5	-2.07	No	NA	
EfM3.048990	-	-1.2	2.0	**2.2**	**3.8**	-5.02	Yes	NA	
EfM3.050590	-	1.2	-1.3	-1.2	**2.1**	1.29	No	XP_013957240	hypothetical protein TRIVIDRAFT_222299 [Trichoderma virens Gv29-8]
EfM3.050840	-	**-2.3**	**-6.7**	**-5.5**	**-4.3**	-1.71	No	CEJ95220	hypothetical protein VHEMI10714 [Torrubiella hemipterigena]
EfM3.051300	-	**2.1**	1.7	1.2	1.6	**6.73**	No	NA	
EfM3.055320	-	1.0	1.5	1.0	1.8	**2.48**	No	CCE29234	related to cytosolic Cu/Zn superoxide dismutase [Claviceps purpurea 20.1]
EfM3.057230	-	-1.6	**-5.8**	-1.6	1.6	**11.90**	No	XP_023425767	uncharacterized protein
EfM3.058140	-	1.2	**2.9**	1.1	**2.6**	1.94	No	CZS94271	uncharacterized protein FFUJ_05161 [Fusarium fujikuroi IMI 58289]
EfM3.060210	-	1.4	1.0	-1.4	**2.8**	5.65	No	NA	
EfM3.061720	-	-1.0	1.8	-1.4	1.0	**-2.35**	No	NA	
EfM3.062700	sspN	-1.3	**-56.3**	1.3	**-37.4**	**-99.84**	Yes	PHH63509	hypothetical protein CDD81_5790 [Ophiocordyceps australis]
EfM3.062880	-	0.0	**-7.0**	1.0	-2.7	-1.43	No	CCE29635	uncharacterized protein CPUR_03482 [Claviceps purpurea 20.1]
EfM3.067730	-	1.5	1.4	1.0	**3.0**	-1.29	No	NA	
EfM3.068330	-	-1.2	**-2.8**	**-2.1**	**-3.5**	**-2.52**	No	NA	
EfM3.068730	-	1.9	1.3	-1.1	1.9	**6.86**	No	NA	
EfM3.069580	-	-1.2	**-3.2**	-1.7	**-5.4**	**-2.52**	No	CCE33891	uncharacterized protein CPUR_07819 [Claviceps purpurea 20.1]
EfM3.072390	-	-1.1	**2.5**	-1.1	**5.1**	**4.81**	Yes	NA	
EfM3.075190	-	**3.0**	1.5	1.5	2.1	-2.10	No	OAA38301	hypothetical protein NOR_06691 [Metarhizium rileyi RCEF 4871]
EfM3.075450	-	-1.5	-2.0	-1.2	**-2.1**	-1.13	No	CCE33525	uncharacterized protein CPUR_07450 [Claviceps purpurea 20.1]
EfM3.075920	-	1.9	**12.6**	1.6	**3.6**	7.69	Yes	CCE31639	uncharacterized protein CPUR_05492 [Claviceps purpurea 20.1]
EfM3.076450	-	2.0	**3.6**	1.4	1.8	**-2.13**	No	NA	
EfM3.077900	-	**2.6**	**2.2**	1.6	1.8	**8.22**	No	CCE29593	uncharacterized protein CPUR_03440 [Claviceps purpurea 20.1]
EfM3.079420	-	-1.0	**-5.7**	1.0	-1.4	1.69	No	XP_018139353	fungal hydrophobin domain-containing protein [Pochonia chlamydosporia 170]

Table S8- List of DEGs in IP ΔvelA-WT comparison which are common with the proposed core set from Eaton et al. [20].

Gene	Fold Change in IP Δ*velA*-WT
EfM3.000810	-3.1
EfM3.005300	-4.1
EfM3.008360	4.4
EfM3.008740	4.0
EfM3.009220	2.8
EfM3.011910	-2.3
EfM3.014350	-2.7
EfM3.016770	-31.2
EfM3.018170	-3.0
EfM3.018180	-2.4
EfM3.019640	-17.8
EfM3.024230	4.9
EfM3.024700	3.4
EfM3.025850	2.6
EfM3.026630	72.4
EfM3.027570	12.5
EfM3.028490	-4.8
EfM3.035410	5.1
EfM3.037040	69.6
EfM3.040190	83.5
EfM3.040200	41.6
EfM3.041530	2.5
EfM3.041550	4.4
EfM3.041770	55.3
EfM3.044630	17.9
EfM3.045520	-4.0
EfM3.048740	4.7
EfM3.048810	2.6
EfM3.048860	6.3
EfM3.050930	9.7
EfM3.056220	-3.4
EfM3.056230	-3.8
EfM3.056240	-4.7
EfM3.057490	26.2
EfM3.057830	-3.4

EfM3.060250	2.3
EfM3.062230	31.5
EfM3.062310	-7.5
EfM3.062330	-3.0
EfM3.062700	-99.8
EfM3.064170	54.6
EfM3.064190	12.2
EfM3.067590	-2.6
EfM3.068120	-4.6
EfM3.072380	2.2
EfM3.072390	4.8
EfM3.077600	9.0
EfM3.079390	4.0
EfM3.081820	5.0

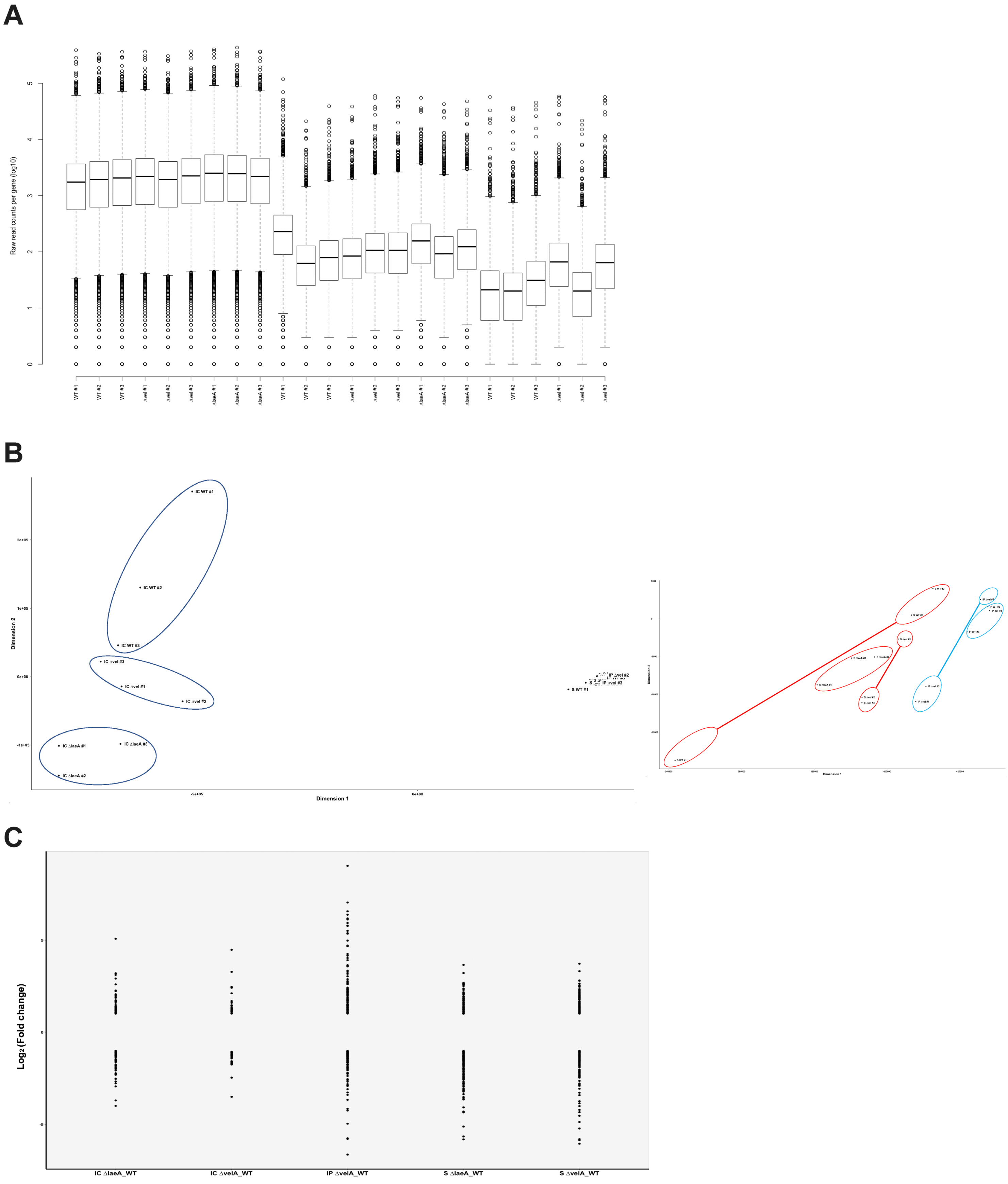

Figure S1- Distribution of number of mapped reads in different samples and fold change in DEGs. A) The bar chart shows the distribution of raw reads mapped per gene. B) Principal component analysis (PCA) of first 1000 highly expressed genes in different samples. C) Fold change distribution of DEGs in different comparisons.

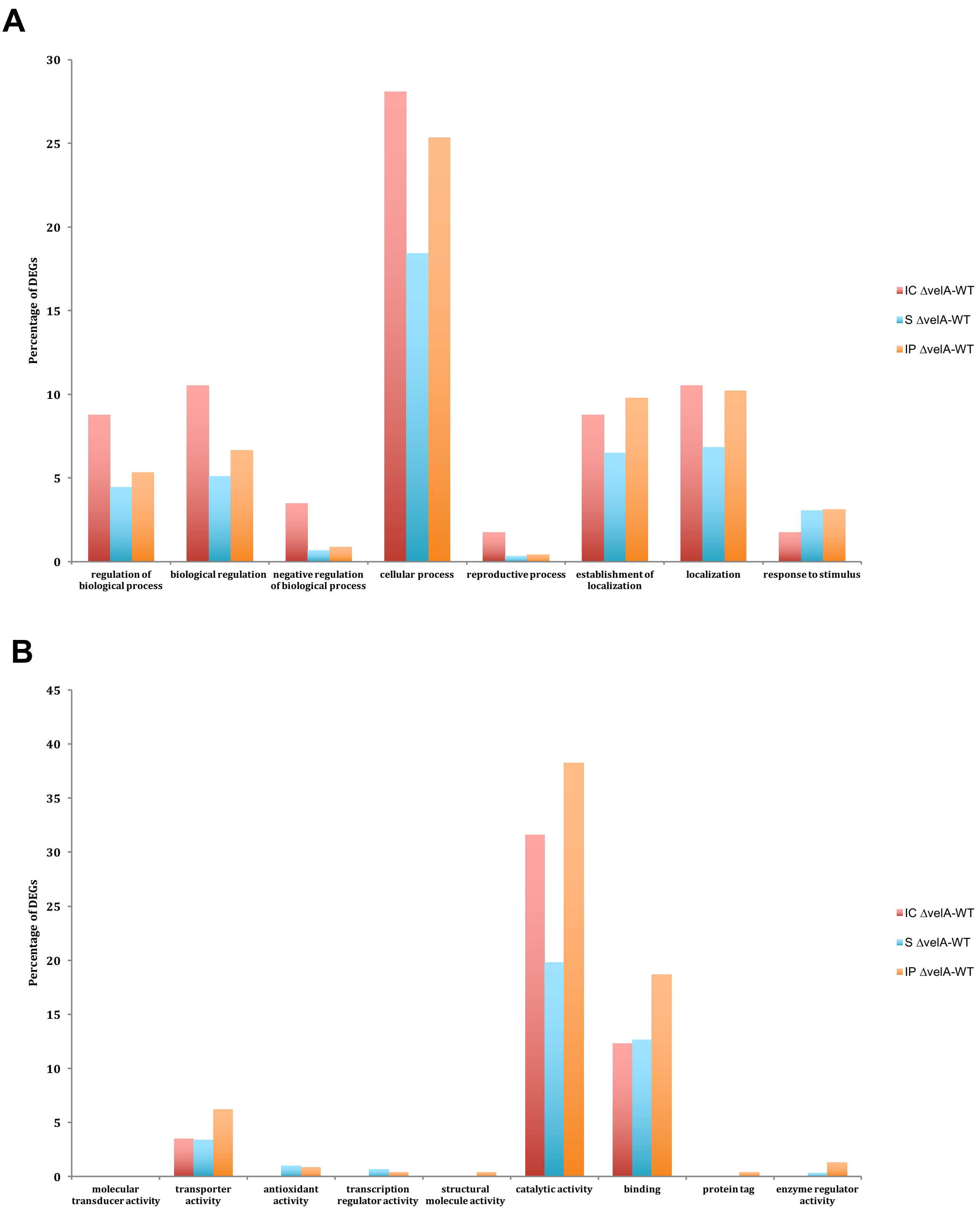

Figure S2- DEGs of different comparisons classified based on primary 'Molecular Function' and 'Biological Process' gene ontology. (a) Bar chart of organised DEGs based on 'Biological Process' GO category. (b) Bar charts of organised DEGs based on 'Molecular Function' GO category. Categories are as follows: GO:0060089 (molecular transducer activity), GO:0005215 (transporter activity), GO:0016209 (antioxidant activity), GO:0030528 (transcription regulator activity), GO:0005198 (structural molecule activity), GO:0003824(catalytic activity), GO:0005488 (binding), GO:0031386 (protein tag), GO:0030234 (enzyme regulator activity).

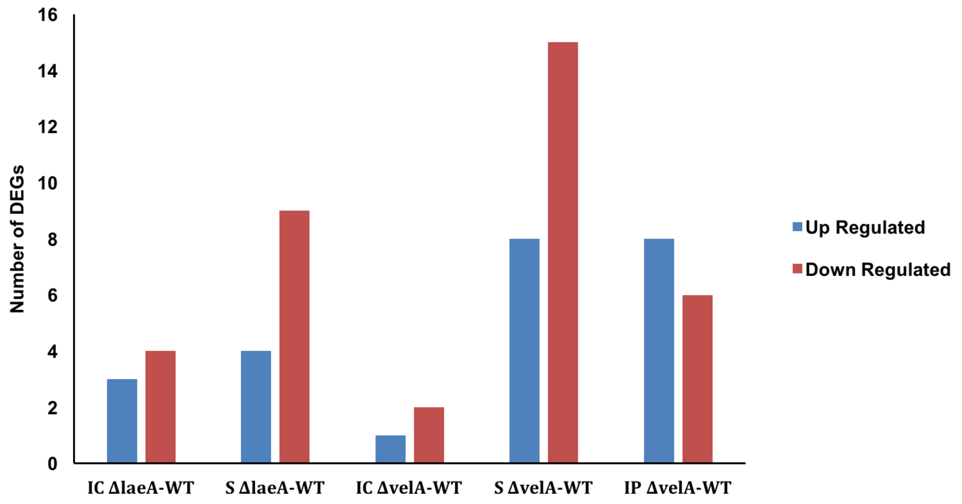

Figure S3- Number of DEGs in different comparisons homologue to CAZyme enzymes in different comparisons.

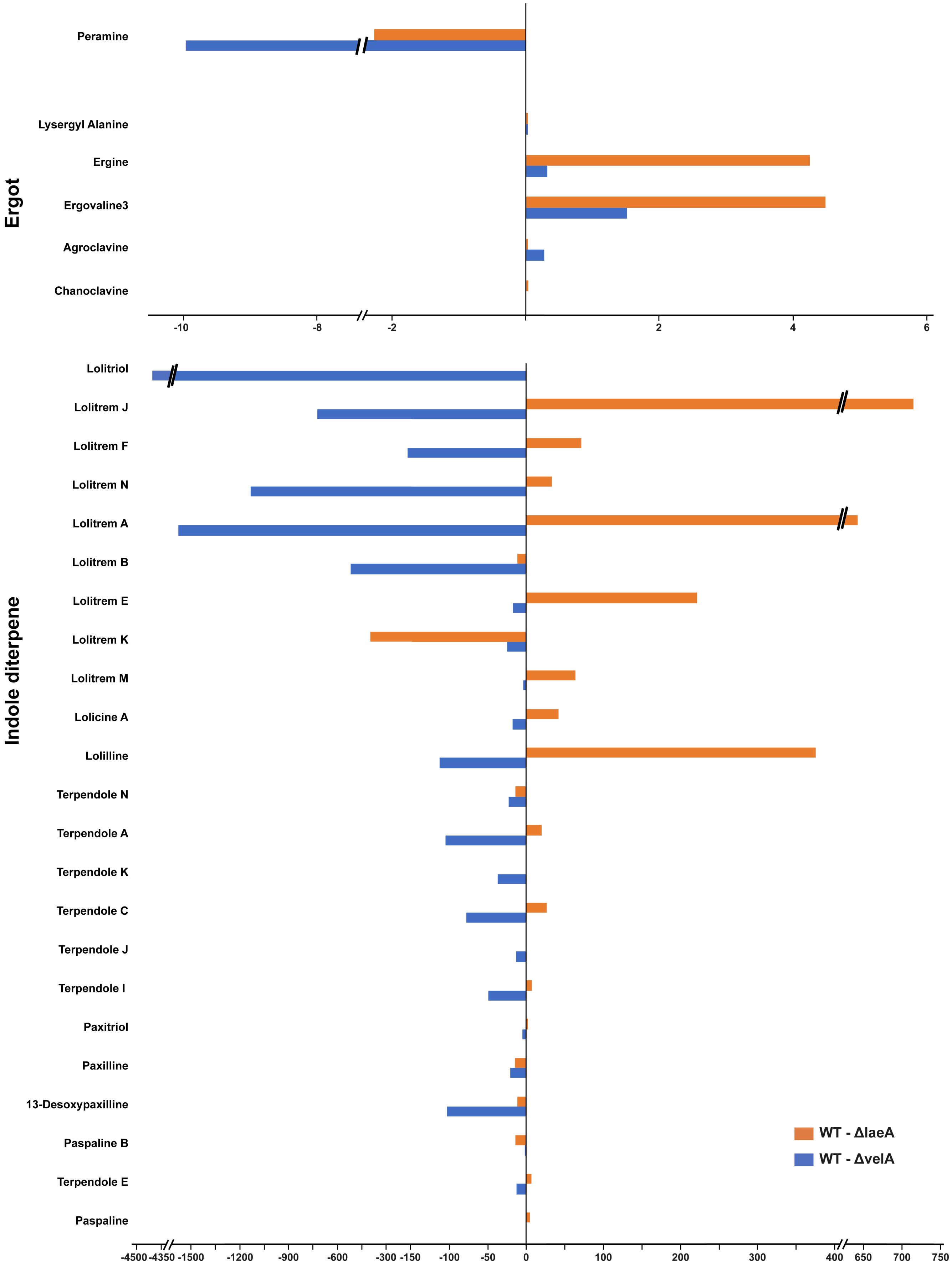

Figure S4- The differences of mean concentration of different alkaloids that produced in the wild type infected plants to the ΔvelA mutant infected plants and the mean concentration differences of wild type infected plants to ΔlaeA mutant infected plants.
